# The influence of narrative medicine on medical students' readiness for holistic care practice: A realist synthesis

**DOI:** 10.1111/medu.70024

**Published:** 2025-08-22

**Authors:** Chien‐Da Huang, Rahma Novita Asdary, Yosika Septi Mauludina, Yufrica Huang, Lynn Monrouxe

**Affiliations:** ^1^ Chang Gung Medical Education Research Centre (CG‐MERC) Linkou Chang Gung Memorial Hospital Taoyuan Taiwan; ^2^ Department of Medical Education and Thoracic Medicine Chang Gung Menorial Hospital, Chang Gung University College of Medicine Taoyuan Taiwan; ^3^ Department of Internal Medicine, School of Medicine Chang Gung University Taoyuan Taiwan; ^4^ School of Health Sciences, Faculty of Medicine and Health The University of Sydney Sydney NSW Australia

## Abstract

**Background:**

The increasing focus on technical skills and efficiency in medical education often overshadows humanistic aspects, creating gaps in preparing clinicians for holistic patient care. Narrative Medicine, integrating storytelling and reflective practices, offers a promising approach to addressing these challenges.

**Objective:**

This study explores how Narrative Medicine interventions influence medical students' readiness for holistic care by examining the mechanisms, contexts and outcomes that underpin its effectiveness.

**Methods:**

A realist synthesis was conducted following Pawson's five‐stage methodology with a two‐phase search strategy across four databases and grey literature. Phase 1 (2008–2018) yielded 10 studies; Phase 2 (2018–2025) added 3, totalling 13. Studies were included if they focused on Narrative Medicine interventions, involved medical students and examined holistic care outcomes. Studies were assessed for credibility, theoretical depth and trustworthiness. Context‐Mechanism‐Outcome (CMO) configurations were iteratively synthesised using ATLAS.ti, ensuring rigour and consistency in accordance with RAMESES standards.

**Results:**

A total of 13 studies, predominantly from U.S. medical schools and hospitals and one from the Netherlands, involving 1158 participants, were analysed. Nine CMO configurations were identified across three contexts: Storytelling Activities, Storytelling Skills and Engaging with Stories. These contexts activated mechanisms such as Deep Observation, Reflection, and Peer Learning, which contributed to outcomes including Enhanced Holistic Patient Connection, Improved Patient Care and Enhanced Self‐Perspective. Notably, Peer Learning showed a weaker linkage to these outcomes, reflecting variability in the effectiveness of Narrative Medicine interventions. Emotional resistance, including avoidance and disengagement, highlighted challenges in implementing Narrative Medicine effectively.

**Conclusion:**

Narrative Medicine enhances relational competence, empathy and reflective practice, aligning with the principles of holistic care. Addressing barriers such as emotional discomfort and variability in outcomes requires adaptable formats and supportive environments. This study underscores Narrative Medicine’s transformative potential in medical education and offers actionable insights for its integration into diverse learning contexts.

## Abstract 2

🚨 New Research Alert! 🚨 How does Narrative Medicine shape medical students' readiness for holistic care? 🩺📖 Using a realist synthesis approach, we uncover key mechanisms, contexts, and outcomes

**Abbreviations**
CMO: Context‐Mechanism‐Outcome
RAMESES: The Realist and Meta‐narrative Evidence Syntheses: Evolving Standards

## 1 INTRODUCTION

Medicine, traditionally regarded as both a science and an art, requires a delicate balance between intellectual rigour and emotional insight. However, the humanistic aspects of medical practice are increasingly sidelined in today's healthcare landscape, which emphasises efficiency, dependability and measurable outcomes.^1^, ^2^ As healthcare shifts toward an industry‐focused model, foundational values such as empathy, individuality and patient‐centred care risk being overshadowed, leading to significant consequences. This shift contributes to rising rates of emotional exhaustion, burnout and diminished professional efficacy among practitioners.^3^ Despite recognition of these challenges, medical education often prioritises technical skills at the expense of relational competence, creating a gap in preparing clinicians for the psychosocial demands of patient care.^4^ This gap contributes to burnout and depersonalisation among practitioners, placing strain on doctor‐patient relationships and leading to decreased patient satisfaction and compromised healthcare outcomes.^3^, ^5^ Addressing this gap is essential, as the diminished focus on humanistic values impacts both practitioners and patients, leading to impersonal, strained interactions and, in some cases, even contributing to violence against healthcare professionals.^6^

To counteract these trends, scholars have advocated holistic models such as Engel's biopsychosocial framework, which integrates biological, psychological and social dimensions of health.^7^ Expanded in 2004, this model emphasises the importance of understanding patients' subjective experiences, promoting relational care and addressing the limitations of purely biomedical reasoning.^8^ Narrative Medicine can be seen as one of the contemporary educational applications of Engel's model, encouraging clinicians to elicit and interpret patients' narratives in ways that incorporate emotional, social and contextual information into care delivery. As articulated by Rita Charon, Narrative Medicine fosters narrative competence, enabling physicians to engage with patients empathetically, reflectively and relationally, thereby restoring meaning and personhood to the clinical encounter.^9^ Recognising this potential, narrative medicine has been introduced into curricula internationally.^10^

However, to date, it remains unclear if and how this approach facilitates students' readiness to engage in holistic practice. Several systematic reviews have examined the use of Narrative Medicine in medical education. These reviews report consistent short‐term benefits, such as improved empathy, communication skills and reflective capacity, but they highlight limited insight into long‐term behavioural or clinical change.^11^, ^12^ Other work has explored its integration in digital contexts, emphasising the need for clearer theoretical foundations and mechanisms of impact.^13^ One review focusing on paediatric education offered useful content summaries but did not examine underlying processes or contextual moderators.^14^ A separate review in oncology settings highlighted promising impacts on clinician wellness and reflection but similarly lacked explanatory depth regarding how narrative practices lead to these outcomes.^15^ Although collectively these studies confirm that Narrative Medicine is valued, they primarily describe programme content and reported outcomes without clarifying how these effects are generated or shaped by different conditions. These gaps in the existing evidence base justify the need for a realist synthesis, which can uncover not just whether Narrative Medicine works, but how and under what conditions it supports holistic learning outcomes.^16^, ^17^, ^18^ We aim to fill this gap by undertaking a realist synthesis to examine what aspects of a narrative medicine intervention impact students' readiness for holistic practice and why. In this study, we position Narrative Medicine not only as our object of inquiry but also as a guiding conceptual framework, shaped by humanistic and developmental theories that inform our programme theory.

### 1.1 Holistic Care

Holistic medicine emphasises treating the “whole person,” encompassing biological, psychological, social and spiritual dimensions of health rather than focusing solely on symptoms. As James Gordon notes, this model contrasts with traditional approaches by prioritising patient empowerment, preventive care and lifestyle changes—key aspects that are especially crucial in managing chronic and geriatric conditions.^19^, ^20^

Holistic care involves comprehensive assessments, promoting patient self‐management and addressing the root causes of illness. Empathy, effective communication and collaboration are central to fostering trust and improving outcomes.^20^, ^21^ These characteristics are integral to building patient‐centred care frameworks, aligning with the increasing complexity of healthcare needs.

Frameworks such as the International Classification of Functioning and the capability approach emphasise autonomy and integrate the biological, emotional and social dimensions of health.^22^, ^23^ In practice, holistic care integrates patients' physical, emotional and social needs, fostering well‐being and enhancing healthcare experiences.^24^, ^25^

### 1.2 Narrative Medicine & Holistic Care

Narrative Medicine bridges biomedical models and humanistic care by incorporating patient stories into clinical practice. It emphasises developing narrative competence—the ability to interpret and respond empathetically to patient narratives—to enhance clinician‐patient relationships, address psychological needs and promote patient‐centred care.^26^, ^27^, ^28^

Pioneered by Rita Charon, Narrative Medicine enhances empathy, reflective skills and bio‐psychosocial understanding through active listening and storytelling.^9^, ^27^ It aligns clinical interventions with patient values and improves communication, making it a vital complement to evidence‐based approaches.^28^, ^29^ Despite challenges in standardising training, its humanistic emphasis and therapeutic potential make Narrative Medicine an essential addition to modern healthcare.^30^, ^31^

As healthcare education seeks ways to bridge the gap between clinical competence and humanistic readiness, Narrative Medicine emerges as a promising approach. Programmes like Narrative Medicine immerse students in patient narratives, fostering empathy, reflective practice and patient‐centred care.^32^ It aligns with holistic care principles by encouraging medical practitioners to view patients as whole individuals, integrating their stories into clinical decision‐making. In this review, we conceptualise holistic care as encompassing both patient‐directed care and the internal development of the clinician. Narrative‐based learning promotes professional identity formation and cultivates the attitudes and behaviours necessary for compassionate, whole‐person care. These internal outcomes are increasingly recognised as vital components of holistic practice.^13^ Programmes such as My Life, My Story have also demonstrated how narrative‐based interventions promote holistic understanding by deepening empathy and reducing ageist attitudes.^33^ Despite these promising outcomes, the specific mechanisms through which Narrative Medicine contributes to holistic care preparedness remain underexplored. Greater insight into these mechanisms is needed to guide curriculum development and adapt interventions to varying educational contexts.

This study aims to explore the influence of Narrative Medicine on medical students' preparedness for holistic care practices. Aligned with the principles of realist research, it seeks to answer: “How do Narrative Medicine approaches impact medical students' readiness for holistic, patient‐centred care?”. By applying a realist review, this study not only evaluates the relative effectiveness of Narrative Medicine interventions but also offers actionable insights for tailoring these interventions to diverse educational and clinical environments, ensuring their alignment with institutional goals and learners' needs.

## 2 METHODS

### 2.1 Realist Synthesis

Realist synthesis is a theory‐driven approach to evidence synthesis rooted in critical realism. It asks, “What works, for whom, in what contexts, how, and why?”, making it well‐suited for complex educational interventions like Narrative Medicine.^34^, ^35^

#### 2.1.1 Purpose and Value of Realist Synthesis

Rather than aggregating outcomes, realist synthesis develops and refines programme theories by examining how specific contexts activate mechanisms that generate outcomes.^36^, ^37^ It offers explanatory insights into why interventions succeed or fail across varying settings.

Although the terms “realist review” and “realist synthesis” are often used interchangeably,^35^, ^37^ we use the term *synthesis* to reflect our aim to construct and refine explanatory programme theories across varied empirical sources.

#### 2.1.2 CMO Configuration

Context–Mechanism–Outcome (CMO) configurations are the central analytic tools in realist synthesis, designed to explain how and why interventions succeed or fail in particular circumstances.^34^ Each configuration links a context (e.g. institutional culture), a mechanism (e.g. learner reflection or emotional insight) and an outcome (e.g. increased empathy or readiness for holistic care).^35^

Unlike conventional tools that measure outcomes in isolation, CMO configurations focus on understanding causal pathways by examining how specific contextual features activate mechanisms that lead to particular outcomes.^36^ This makes them especially useful for evaluating complex interventions like Narrative Medicine, where effects may vary depending on institutional settings, learner backgrounds and instructional approaches.^37^

In this study, CMO configurations served as the guiding framework for both data extraction and theory refinement. They were operationalised through systematic coding in ATLAS.ti and explored through co‐occurrence analysis to identify causal patterns across multiple studies.^35^ These configurations were not predetermined but refined iteratively as data were analysed.

#### 2.1.3 Methodological Rigour and Adaptability

To ensure rigour, we followed the RAMESES publication standards for realist synthesis,^35^ which emphasises transparency, relevance and conceptual clarity. Our team conducted iterative discussions, reflexive analysis and peer debriefing throughout each stage of synthesis.

Although realist synthesis does not involve original data collection, it integrates findings from studies that use a variety of designs, including qualitative interviews, quantitative surveys and implementation reports. This diversity allows for triangulation at the level of evidence integration, where different forms of data are compared to test, support or refine emerging programme theories. In realist synthesis, triangulation occurs at the level of mechanisms across studies. This means we examine whether similar mechanisms are reported in different contexts or study designs, rather than comparing individual participant responses. This approach to triangulation strengthens the explanatory power of CMO configurations by assessing whether consistent patterns of causality are observed across varied settings. It enhances both the credibility and the practical utility of the resulting programme theories.^36^, ^37^

### 2.2 Study Design

Our study design follows the five‐stage approach adapted from Pawson's methodology, as outlined in our registered protocol,^38^ serving as a foundational guide for this research process.

#### 2.2.1 Stage 1: Locate Existing Theories

To construct an initial programme theory, we first reviewed theoretical frameworks relevant to Narrative Medicine and holistic care. Engel's biopsychosocial model^7^ provided an essential foundation by integrating biological, psychological and social dimensions of illness and care, offering a counterpoint to reductionist biomedical paradigms. In parallel, we treated Narrative Medicine not only as the subject of inquiry but also as a guiding conceptual framework. At its core, Narrative Medicine defines narrative competence as the ability to recognise, interpret and be emotionally moved by patients' stories, which is considered a prerequisite for humanistic, person‐centred care.^26^ This framing closely aligns with the aims of holistic medical education, particularly in cultivating learners' relational and reflective capacities.

We also drew on models of professional identity formation and reflective practice, which synthesise a range of theoretical influences to explain how learners develop internal capacities and relational behaviours in clinical settings.^39^, ^40^ These models helped us interpret how narrative‐based interventions might influence personal development and support readiness for holistic care.

While we recognise that in Narrative Medicine is a broad and evolving approach rather than a single unified theory, we adopt its foundational premise—centering narrative competence as a core educational mechanism—as the anchor for our programme theory. We also acknowledge variations in Narrative Medicine implementation across contexts and explore how these differences interact with contextual and mechanistic factors throughout the synthesis.

This framing helped refine the scope of our review and provided sensitising concepts to guide early theory development, consistent with realist principles of iterative theory building through data engagement.

#### 2.2.2 Stage 2: Search Strategy

Our search strategy followed a two‐phase realist approach, as outlined in our published protocol.^38^ In Phase 1, we conducted a systematic search in four major databases—Web of Science, Medline, Scopus and Embase—using terms such as “narrative‐based medicine,” “narrative medicine” and “holistic care,” covering publications between January 2008 and September 2018. This date range was predefined in our PROSPERO registration (CRD42018115447) to ensure feasibility for theory development during our realist synthesis. To update the evidence base, Phase 2 involved a targeted supplementary search (October 2018 to June 2025).

#### 2.2.3 Stage 3: Study Selection

**FIGURE 1:**
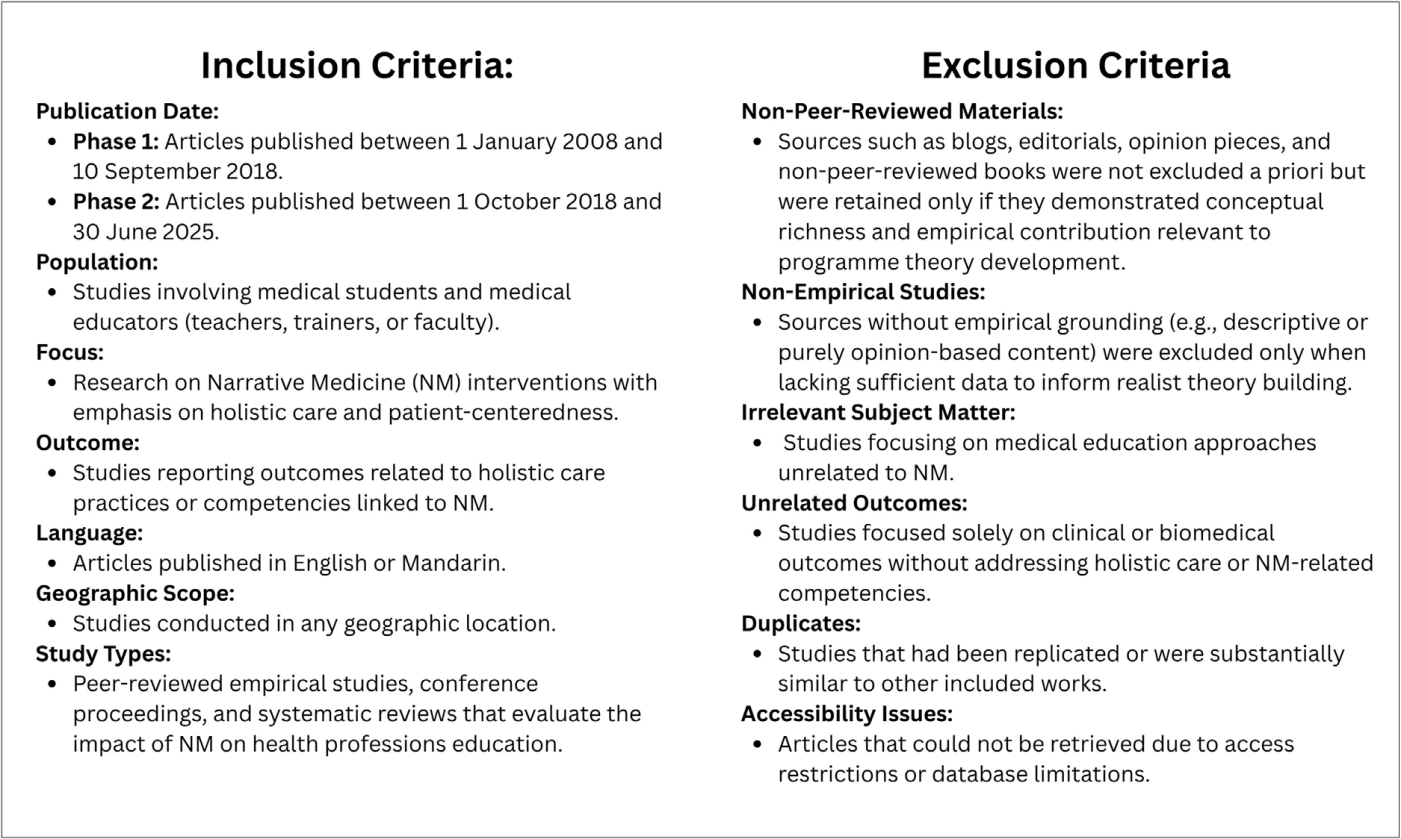
Inclusion and Exclusion Criteria.

Researchers independently screened articles using Endnote, with an initial screening of titles and abstracts conducted by YH and re‐screened by CDH and YSM. References that did not addressing both Narrative Medicine and holistic care were excluded, and selection was limited to English and Mandarin due to team language expertise. Inclusion and exclusion criteria are detailed in Figure 1. After removing 484 duplicates, 557 records were screened at title/abstract level. Of these, 103 articles were retained for full‐text review in Phase 1. Following the assessment of criteria (Figure 1), 10 studies were included in the final realist synthesis.

**FIGURE 2:**
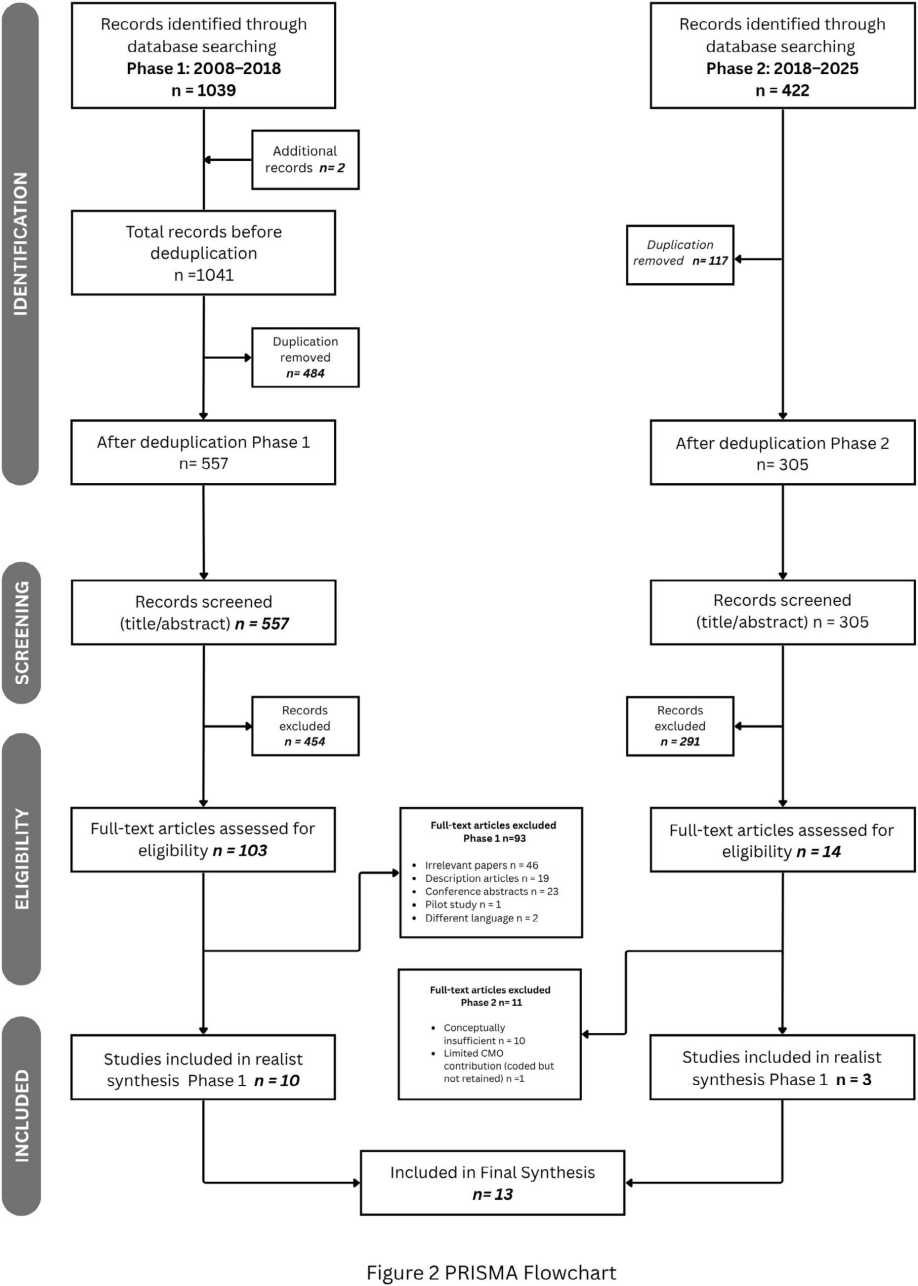
PRISMA Flowchart.

In Phase 2, 14 additional full‐text articles were assessed. Four of these were included for the CMO development with 10 being excluded due to limited conceptual grounding. A further study was subsequently excluded due to insufficient groundedness of the CMO configurations identified. These distinctions are illustrated in the combined PRISMA flow diagram (see Figure 2).

Grey literature sources, including policy documents and conference proceedings, were also reviewed at the full‐text stage in accordance with our published protocol. Non‐peer‐reviewed materials were not excluded a priori; however, inclusion was contingent on meeting our prespecified thresholds for conceptual richness and empirical contribution to theory development. None of the grey literature sources met these thresholds. This outcome reflects the application of realist review principles, which encourage broad inclusion during initial theory development while emphasising analytic rigour during synthesis.^36^, ^37^

#### 2.2.4 Stage 4: Data Extraction and Quality Appraisal

Primary data extraction was conducted by LVM and RNA, who recorded details on authorship, aims, methods, findings and study populations. Articles were assessed for their contribution to the programme theory, categorised by conceptual richness (high, moderate or low) and appraised for credibility, theoretical depth and trustworthiness. Conceptual richness was defined by the presence of multiple CMO components and evidence supporting causal explanations rather than surface‐level descriptions.

All studies were managed and coded using ATLAS.ti software. Regular team discussions among CDH, RNA, LVM, YSM and YH ensured consensus and resolved discrepancies. Through this process, key Context, Mechanisms and Outcomes (CMOs) were identified and defined, providing a comprehensive framework for coding. Methodological rigour was maintained by adhering to PRISMA guidelines.^41^ For example, we followed structured processes for systematic data extraction, ensured transparent reporting of the study selection process and employed a PRISMA flow diagram (Figure 2) to illustrate the inclusion and exclusion process.

#### 2.2.5 Stage 5: Data analysis and synthesis

To explore key contexts and mechanisms influencing students' readiness for holistic care practice, LVM and CDH synthesised Stage 4 data to refine the programme theory.^42^ Findings were systematically tested and refined using conceptual tools^38^: juxtaposing, reconciling, adjudicating, consolidating and situating within CMO configurations. This process was conducted in ATLAS.ti through several analytical steps: Contexts, Mechanisms and Outcomes were identified through close reading of the articles, and excerpts were selected and coded, ensuring that all three CMO aspects were coded together where possible. This meant that excerpts typically comprised narratives of events where all three Contexts, Mechanisms and Outcomes constructs are typically present. Phase 1 articles were analysed first, then Phase 2 articles were added at a later stage to the database and analysed in the same manner.

The data were analysed in ATLAS.ti for ‘co‐occurrences’ between Contexts and Mechanisms. The analytic process was abductive and retroductive, integrating empirical findings with theoretical reasoning to refine causal explanations. Subsequently, these Context‐Mechanism were developed into ‘smart codes’ which were analysed for co‐occurrences with Outcome codes. This formed our collective CMO ‘smart codes’ facilitating our mapping of causal patterns in the data (our final CMO configurations) and to understand to what extent these were grounded in the data. Groundedness, a metric in ATLAS.ti, indicates how frequently a code appears across the dataset.^43^ Only articles with groundedness scores above four were considered for the synthesis; others were excluded due to limited theoretical relevance. The analysis and writing refinement of CMO configurations were conducted by LVM and RNA, ensuring that results were systematically reported following RAMESES standards (as detailed in Supplementary 2) for realist syntheses.^35^

This staged, theory‐driven approach aligns with realist synthesis principles, which emphasise iterative searching and purposive inclusion to refine programme theories rather than achieve exhaustive coverage.^35^, ^44^ The original 2018 cut‐off ensured analytic manageability, whereas the updated supplementary search (to 2025) confirmed the continued validity of our CMO configurations.

## 3 RESULTS

A total of **1463 records** were identified across two phases of database searching and manual screening. After removing **862 duplicates** and excluding **745 records** during title and abstract screening, **117 full‐text articles** were assessed for eligibility. **Thirteen studies** met the inclusion criteria and were included in the final realist synthesis, comprising 10 from Phase 1 ^16,18,33,45–51^ and three from Phase 2.^33^, ^45^, ^46^ One additional Phase 2 study was coded during CMO development but was excluded from final synthesis due to limited conceptual grounding.^47^ Detailed characteristics of included studies are provided in Table S1. Most studies were conducted in the United States, with one from the Netherlands. Nine studies took place in university settings, two in combined university and hospital environments, and two in hospital‐only settings. Only one study involved multiple sites. Collectively, these studies involved 1158 participants, 645 from Phase 1 and 513 from Phase 2, (median: 42 per study; IQR: 17–130) and employed diverse methodologies, including mixed methods were used in 61.5% of studies, and thematic analysis was the dominant analytical technique reflective writing, focus groups and storytelling, to foster empathy, self‐awareness and narrative competence (See Table 1).

**TABLE 1:** Characteristics of Narrative Medicine and Realist Evaluation Studies.

Characteristic	n	Percentage	Studies Included
**Intervention**			
‐ Narrative medicine electives	3	23.08	(Arntfield et al., 2013, Miller et al., 2014, Stumbar et al., 2023)
‐ Storytelling	2	15.38	Morgan et al. (2025); Chretien et al. (2015) (Chretien et al., 2015, Morgan et al., 2025)
‐ Workshops	4	30.76	(DasGupta and Charon, 2004, Green, 2015, Leijenaar et al., 2023, Lemay et al., 2017)
‐ Writing exercises	4	30.77	(Brown et al., 2015, Garrison et al., 2011, Levine et al., 2008, Wesley et al., 2018)
**Methodological Approach**			
**Mixed Methods (8 studies)**			
‐ Likert‐type Scale Assessments	4	30.80	(Green, 2015, Lemay et al., 2017, Morgan et al., 2025, Stumbar et al., 2023)
‐ Thematic Analysis	8	61.50	(Brown et al., 2015, Chretien et al., 2015, Green, 2015, Leijenaar et al., 2023, Lemay et al., 2017, Miller et al., 2014, Morgan et al., 2025, Stumbar et al., 2023) (Note: Thematic Analysis appears in both Mixed and Qualitative categories based on the original data, so the total count for thematic analysis is 12. Here, it is counted for each time it appears within a Mixed Methods study.)
‐ Focus Groups	4	30.80	(Brown et al., 2015, Chretien et al., 2015, Miller et al., 2014, Morgan et al., 2025)
‐ Patient Interviews	1	7.70	(Chretien et al., 2015)
‐ Grounded Theory Analysis	1	7.70	(Brown et al., 2015)
‐ REFLECT rubric scoring	1	7.70	(Leijenaar et al., 2023))
‐ Open‐Ended Surveys/Questions/Forms	1	7.70	(Lemay et al., 2017)
**Qualitative (5 studies)**			
‐ Thematic Analysis	4	30.80	(DasGupta and Charon, 2004, Garrison et al., 2011, Levine et al., 2008, Wesley et al., 2018) (Note: Thematic Analysis appears in both Mixed and Qualitative categories based on the original data, so the total count for thematic analysis is 12. Here, it is counted for each time it appears within a Qualitative study.)
‐ Focus Groups	1	7.70	(Arntfield et al., 2013)
‐ Open‐Ended Surveys/Questions/Forms	3	23.10	(Arntfield et al., 2013, DasGupta and Charon, 2004, Wesley et al., 2018)
‐ Grounded Theory Analysis	1	7.70	(Arntfield et al., 2013)
‐ Reflective Writing (as data)	2	15.40	(DasGupta and Charon, 2004, Garrison et al, 2011, Levine et al, 2008)
‐ Content Analysis (of writing)	1	7.70	(DasGupta and Charon, 2004)
**Quantitative (0 studies)**			
‐ None	0	0	(No purely quantitative studies in this dataset)
**Patient Involvement**			
‐ Yes	4	30.80	(Brown et al., 2015, Chretien et al, 2015, Garrison et al., 2011, Morgan et al., 2025)
‐ No	9	69.20	(Arntfield et al., 2013, DasGupta and Charon, 2004, Green, 2015, Leijenaar et al., 2023, Lemay et al., 2017, Levine et al., 2008, Miller et al., 2014, Stumbar et al., 2023, Wesley et al, 2018)
**Number of Participants**			
‐ Small cohorts (10–20)	5	38.50	(Arntfield et al., 2013, DasGupta and Charon, 2004, Miller et al, 2014, Stumbar et al, 2023, Wesley et al, 2018)
‐ Medium cohorts (30–50)	4	30.80	(Chretien et al., 2015, Garrison et al., 2011, Green, 2015, Levine et al., 2008)
‐ Large cohorts (>100)	4	30.80	(Brown et al., 2015, Leijenaar et al., 2023, Lemay et al., 2017, Morgan et al, 2025)

### 3.1 CMO Analysis

Through our analysis, we identified four key mechanisms—Reflection, Deep Observation and Deeper Patient Engagement, which positively impact holistic care and professional development, and Emotional Avoidance, which led to a negative outcome. The three positive mechanisms were well‐grounded in the data with numerous examples across the studies. These mechanisms, activated across the three contexts of Storytelling Activities with Patients, Storytelling Skills and Engaging with Stories, contributed to the diverse yet related outcomes of Holistic Patient Connection, Patient Care, Ability to Work with Colleagues and Self‐Perspective. However, we also identified Emotional Avoidance as a mechanism that led to a negative outcome: Disengagement. Additionally, we identified one further mechanism, Peer Learning, which we defined as *“learning from one another through interaction and reflection, rather than rigidly adhering to specific teaching content or hierarchical student‐teacher relationships.”* This mechanism was strongly grounded in the context of Engaging with Stories; however, its outcomes were weakly linked (i.e., coded fewer than three times) across various domains, including Holistic Patient Connection, Patient Care and Self‐Perspective.

**FIGURE 3:**
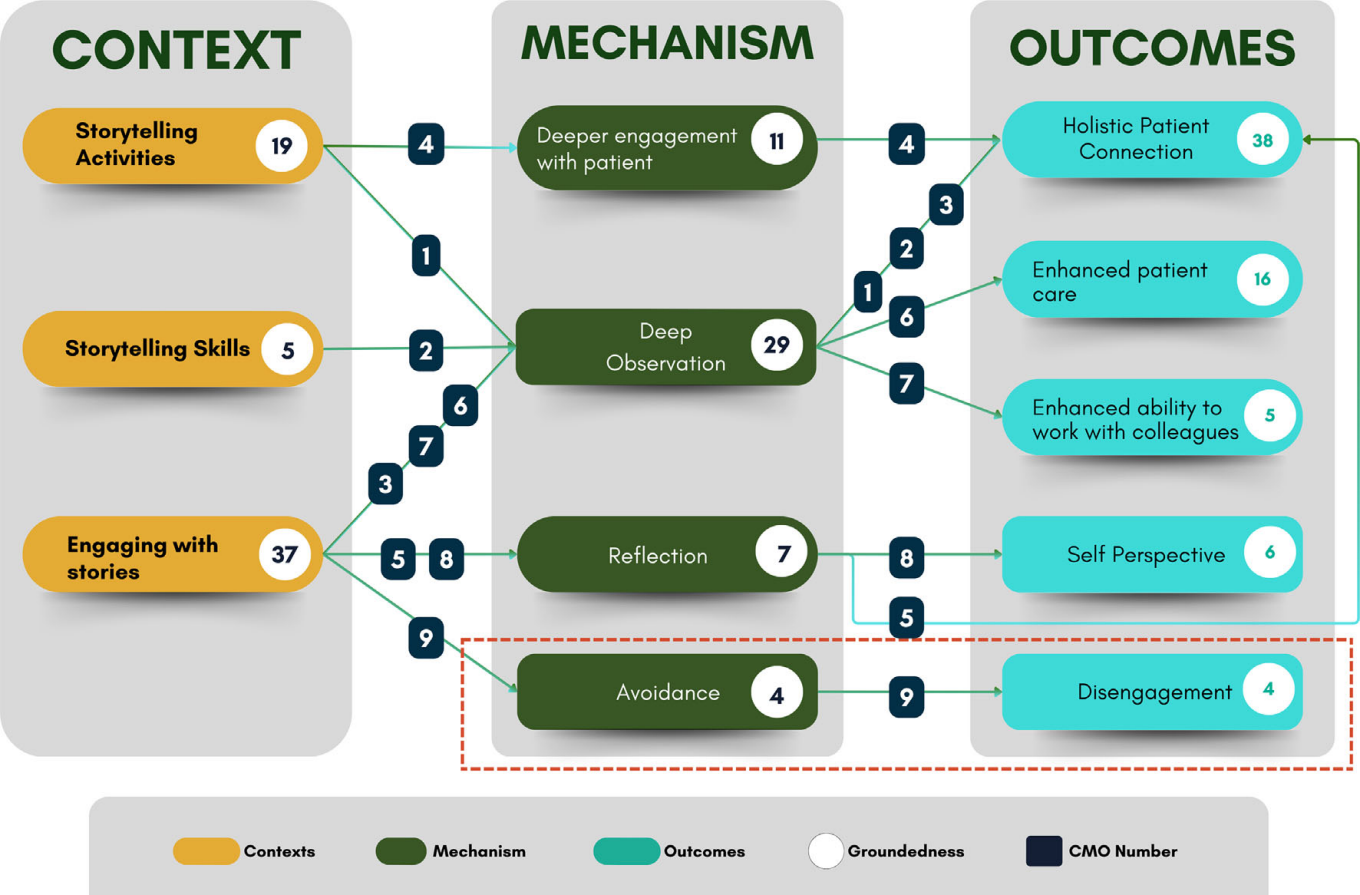
Relationship between Contexts, Mechanisms and Outcomes in Narrative Medicine Practice. [Color figure can be viewed at wileyonlinelibrary.com]

Our key findings are detailed through nine CMO configurations (note, the literature search in Phase 2 served to further underpin some of our CMO configurations, but failed to identify any new ones). Each CMO is defined in Table 2, with a schematic overview of how they link together being summarised in Figure 3, and illustrative examples of each CMO configuration being presented in Table 3. We now present these nine CMO configurations below, grouping them by the outcomes identified.

**TABLE 2:** Operational Definitions and Groundedness of the identified CMO configurations.

Code	Groun‐dedness*	Definition
Context		
Engaging with Stories	37	This includes facilitated reading activities (poems, fiction, non‐fiction, doctors' accounts of practice, film and/or working with illness narratives), writing exercises (including illustrated writing and reflective writing) and exploring their own/peers illness narratives
Storytelling Activities with Patients	19	Here, students spend time listening to patients' stories and develop their deep listening skills/reporting by recounting them back (verbally or written)
Storytelling Skills	5	This context is about the direct and purposive development of the skills of storytelling through understanding what narratives are and the different elements of storytelling (e.g. metaphor use)
Mechanisms		
Deep Observations	29	Understanding narrative through deep observation in both written and oral formats: Including attending to metaphors, word choices, emotional content and non‐verbal details
Deeper Engagement with Patient as Person	11	Engaging a sense of understanding and responsibility; developing a sense of human connection
Reflection	7	The process of critically examining and interpreting someone's experiences, thoughts and emotions.
Avoidance	4	Disengagement from the activity either in the belief it is futile or sometimes due to discomfort
Outcomes		
Enhanced Holistic Patient Connection	38	This centres on fostering a deeper, more empathetic understanding of patients, emphasising their individuality beyond their medical condition. It involves recognising patients as individuals with unique lived experiences, ensuring culturally sensitive interactions and avoiding stereotypes. Trust is built by including patients in decision‐making processes, valuing their perspectives and acknowledging that healthcare providers do not always know best
Enhance Patient Care	16	Includes using narrative and story sharing to involve the patient in their care and recovery plan, or having a more patient‐centred approach to diagnosis and treatment; Enhanced clinical diagnosis; Enhanced observation skills
Enhanced Self Perspective	6	Feeling better about oneself; self‐care; Enhanced self‐awareness; maintaining a sense of self
Enhanced Ability to Work with Colleagues	5	Greater understanding of peers
Disengagement	4	Difficulty in considering the patient perspective, feeling empathy or engaging in the process

* Groundedness refers to the number of times each construct was coded across the data.

**TABLE 3:** Summary of Context‐Mechanism‐Outcome (CMO) Configurations in Narrative‐Based Practices.

No	Context	Mechanism	Outcome	Example Quote	Contributing Study
1.	Storytelling Activities with Patients	Deep Observation	Holistic Patient Connection	Some students experienced a transformation in their patients after the activity. “By the end of the activity he really seemed … a different person because he … had more understanding and willingness to cooperate.”(Chretien et al, 2015, p.1026)	(Chretien et al, 2015, Garrison et al, 2011)
2.	Storytelling Skills	Deep Observation	Holistic Patient Connection	“narrative training to improve the capacity to understand patients, extend empathic care and communicate well with patients and families and other medical providers. One thought his or her training supported the use of silence, healing touch and to say, “I do not know.” Another recognised that medical documentation can include social and emotional dimensions in retrospect”(Arntfield et al, 2013, p.8)	(Arntfield et al, 2013, Brown et al, 2015, Levine et al, 2008)
3.	Engage with Stories	Deep Observation	Holistic Patient Connection	“Examining patient encounters through narrative writing and editing, as well as through listening to other participants share stories about experiences with patients, helped achieve a deeper understanding of the lived experiences of patients”(Lemay et al., 2017, p.358)	(Arntfield et al, 2013, Lemay et al, 2017, Miller et al, 2014)
4.	Storytelling Activities with Patients	Deeper Engagement with Patient as Person	Holistic Patient Connection	She [student] wrote a little thing about me [patient] … it really touched me, deep, you know, and I cannot think of anybody, at least in a couple decades, who has helped me with such high esteem. You know, when I'm here and I'm not in my best of shape or anything … that did not matter to her, she saw me as an individual and saw me as having self‐worth [tears up] (Chretien et al, 2015, p.1027)	(Chretien et al, 2015, Garrison et al, 2011)
5.	Engaging with Stories	Reflection	Holistic Patient Connection	Finally, students comment on how creating a comic about a formative experience provides an opportunity for reflection that influences how they intend to relate to patients in the future. The course made me revisit my experience with Bell's palsy … and taught me the value of never minimalising a patient's condition, no matter how small. The story I used for my own graphic narrative taught me a lot about this— particularly about making assumptions that you know what is best for a patient” (Green, 2015, p.777)	(DasGupta and Charon, 2004, Green, 2015, Stumbar et al., 2023)
6.	Engaging with Stories	Deep Observation	Enhanced Patient Care	Students felt they developed an increased capacity to understand and empathise with patients and “see them as a person or narrative other than an illness.” Many emphasised the “humanity” of patients and the importance of interactions with them: “discussions like these help to reiterate that medicine is about person to person contact” and “helps to remind you that while you are having just another day at the hospital… it's probably the worst day of that person's life, or one of them. (Arntfield et al, 2013, p.8)	(Arntfield et al, 2013, Chretien et al, 2015, Garrison et al, 2011)
7.	Engaging with Stories	Deep Observation	Enhanced Ability to Work with Colleagues	The applicability of sessions to interactions with other physicians was also reported, although not as frequently: “we also have to see our colleagues as more than, you know, your consulting nephrologist… I mean, that's a person as well, and how you interact with them is important.…” (Arntfield et al, 2013, p.5)	(Arntfield et al, 2013, Levine et al, 2008)
8.	Engaging with Stories	Reflection	Enhanced Self‐Perspective	My course [focused on] writing your thoughts … and how that can be therapeutic, in regards to the patient and also the person that is administering to the patient— especially something that might be very difficult for you to deal with, such as death of the patient … and the idea that maybe writing that out might help you to … get your thoughts and feelings out and organise what, I guess, in the end you thought about the experience (Miller et al, 2014, p.9)	(Arntfield et al, 2013, Levine et al, 2008, Miller et al, 2014, Leijenaar et al, 2023, Stumbar et al, 2023)
9.	Engaging with Stories	Avoidance	Disengagement	“It just sometimes seems very contrived to me … where we were trying to … really looking at something, like, you need to look at the patient this way. And clearly, yes, you need to look at the patient that way. But I think there was so much more you could have gotten out of it, that could not be explained that way. I thought it was kind of cheapening it a little” (Miller et al, 2014, p.6)	(Brown et al, 2015, Chretien et al, 2015, Miller et al, 2014, Morgan et al, 2025)

### 3.2 Outcome: Enhanced Holistic Patient Connection

Enhanced Holistic Patient Connection—which focuses on fostering empathy for patients by recognising their individuality, respecting cultural nuances, avoiding stereotypes and including them in decision‐making to build trust—was the most frequently identified outcome featuring in five of the ten CMO configurations and supported by evidence in eight of the thirteen articles.^16^, ^46^, ^48^, ^49^, ^50^, ^51^, ^52^, ^53^ Furthermore, within those five CMO configurations, all three contexts—Engaging with Stories, Storytelling Activities with Patients and Storytelling Skills—were linked with the mechanism of Deep Observation in producing this outcome. Notably, Deep Observation was identified as a core mechanism across six articles,^16^, ^48^, ^50^, ^52^, ^53^, ^54^ and is defined as “*the process of understanding narrative through deep observation in both written and oral formats”*. This includes interpreting deeper meanings by attending to metaphors, word choices, emotional content and non‐verbal details in patient stories.

**CMO #1:** **“In the context of Storytelling Activities with Patients, the mechanism of Deep Observation leads to the outcome of Enhanced Holistic Patient Connection”**

Here, in the context of listening to patients narrate their illness, and students sharing their re‐storying and interpretations of those narratives, both patients and students report a sense of connectedness due to students' developing narrative skills. Patients reported to students that they felt understood and appeared to become more open to sharing previously unspoken parts of their stories. Students observed that patients appeared to change during the process, describing it as ‘very therapeutic’ with patients being more willing to ‘cooperate’, and with a sense of ‘closeness and trust’ was established, they appeared more open to sharing and talking: “… *it was very therapeutic … by the end of the session he said that he would start taking the meds again*.”^46, p1026^ Students themselves reported feeling that they knew patients better, felt privileged that patients had shared their stories and reported a greater sense of connection and understanding.

CMO #2: “In the context of Storytelling Skills, the mechanism of Deep Observation leads to the outcome of Enhanced Holistic Patient Connection”

In this situation, the context of narrative training was thought to enable students and junior residents to achieve a greater connection between themselves and patients through their newly developed narrative interpretive skills. These skills facilitated aspects such as empathetic care, understanding that all patients are different and avoidance of stereotyping. Additionally, they fostered cultural sensitivity and deeper understanding of patients as well as the use of subtle communication methods (e.g. silence, non‐verbal methods) to promote deeper connections: “*these junior residents identified their skill in story‐telling and story‐listening to be tools in the work of medicine itself. They recognised the centrality of story‐telling in their daily interactions with peers, superiors, patients, and families*”.^7^, ^16^

CMO #3: “In the context of Engaging with Stories, the mechanism of Deep Observation leads to the outcome of Enhanced Holistic Patient Connection”

Students reported that through the acts of reading, writing and editing a range of story‐based genres (e.g. poems, fiction, non‐fiction, narrative photography, visual arts, doctors' accounts of practice and/or illness narratives) – alongside the telling of, and listening to, stories with each other – they “*have a large bag of tools*”,^16^ ^p.5^ for better connecting with patients. Patience, confidence, empathy’ and being a better listener with the ability to understand patients' narratives helped them achieve a deeper understanding of patients' lived experiences and relate to them more: “*being able to understand narrative will make me a better listener and a better provider”* and *“to listen to what a narrative is telling you about the person's situation and the way they feel about the illness*… *helps you take care of someone well*.”,^16^ ^p.5^

CMO #4: “In the context of Storytelling Activities with Patients, the mechanism of Deeper Engagement with Patient as a Person leads to the outcome of Enhanced Holistic Patient Connection”

Here, the context of Storytelling Activities with Patients impacted on students' understanding that their patient was not just an illness or a problem to be solved, but that they are a person with their own personal history (not just clinical history) ‐ “*letting my patient speak for himself as a person and not as a diagnosis*”,^50^ ^p.87^ – thereby facilitating students seeing patients in a “*different light*”,^50^ ^p.87^ compared to when they first met them in a clinical context. Some students also reported noticing patients' emotional reactions to hearing their stories recounted back to them, while others described developing a heightened sense of responsibility, and some reported a greater sense of connection: “*Many students described their experience of increased connection and therapeutic alliance with their patients. One student noted that the “personal nature of the interview helped dispel some of [the patient's] anxiety about her health care providers”*.,^50^ ^p.87^ It was not only students who reported an enhanced connection, patients also commented on this (see excerpt in Table 3).

CMO #5: “In the context of Engaging with Stories, the mechanism of Reflection leads to the outcome of Enhanced Holistic Patient Connection”

The mechanism of Reflection, as the process of critically examining and interpreting someone's experiences, thoughts and emotions, was activated within the context of Engaging with Stories, where students interacted with patient narratives through reflective writing exercises and narrative reconstructions. This activity created a setting for introspection, enabling students to gain a deeper understanding of patient experiences. This process directly contributes to the outcome of enhanced holistic patient connection, fostering a *“sense of regard and empathy for patients”*.,^49^ ^p.355^ Projecting this into the future was also a factor, for example “*I have found that it is truly important to reflect on your patients as people and think about how the interactions went. I have thought about how my personal feelings play into my professional relationships. I think I will continue to write about my patients when I go into residency*”,^46^ ^p.44^.

### 3.3 Outcome: Enhanced Patient Care

Not only does the mechanism of Deep Observation in the context of developing Storytelling Skills lead to Enhanced Holistic Patient Connection, but we also found evidence that it leads to Enhanced Patient Care. For this outcome, we have one CMO configuration, evidenced through three of the thirteen articles in our synthesis.^16^, ^48^, ^50^

CMO #6: “In the context of Engaging with Stories, the mechanism of Deep Observation leads to the outcome of Enhanced Patient Care”

There was a strong belief among students that the skills of deep observation gained through engaging with stories positively impacts their future practice as physicians, making them better listeners and providers: *“listening to what a narrative reveals about a person's situation and their feelings about the illness… [which] helps you take care of someone well”*.,^16^ ^p.5^ Even within the short timeframe of a patient encounter, some students commented that they were noticing small details that otherwise they would have missed, “*One also found increased skill in interpreting patients' clinical history and unraveling their chief complaints*”.,^16^ ^p.7^ Furthermore, the relationship between developing Enhanced Holistic Patient Connection through Deep Observation (CMO #3) was found to lead to improved clinical outcomes: “*It ended up improving his care later on because I was able to sort of level with him on things to get him the appropriate care he needed*”.,^48^ ^p.1027^

### 3.4 Outcome: Enhanced Ability to Work with Colleagues

Beyond the anticipated benefits for patients and patient care, the mechanism of Deep Observation had an unanticipated impact on students' interpersonal relationships, fostering a more harmonious and collaborative learning environment.^16^, ^55^

CMO #7: “In the context of Engaging with Stories, the mechanism of Deep Observation leads to the outcome of Enhanced Ability to Work with Colleagues”

Here, students began to see peers and seniors in a different light (see excerpt in Table 3). They also became more aware of how others might perceive them and developed a greater awareness and understanding of how their Narrative Medicine training *“will help to recognise chaos narratives of over‐stressed, overworked peers”*.,^16^ ^p.5^

### 3.5 Outcome: Enhanced Self‐Perspective

Another unanticipated outcome of the narrative medicine approach is its impact on students' self‐perspectives. Five of the thirteen articles in our study evidenced this aspect.^16^, ^45^, ^46^, ^53^, ^55^

CMO #8: “In the context of Engaging with Stories, the mechanism of Reflection leads to the outcome of Enhanced Self Perspective”

Within the context of Engaging with Stories, the mechanism of Reflection significantly impacted students' relationships with themselves. Students described the reflective process as being “*therapeutic*”, enabling them to cope with challenging situations such as patient death (See Table 3)^53^ while also helping them organise their thoughts and feelings. Additionally, students reported being able to develop their own self‐awareness, understanding themselves better and feeling more positive about themselves through this understanding: *“This study allowed me to… be an observer of myself this last year. In fact, a lot of the negative and positive ways that I've felt this last year are easier to remember since I've had to recall them over the months. I've become a fan/rooter for myself by the end… (like it's a sporting event)!”*,^55^ ^p.728^ Other key aspects of themselves and their lives were highlighted, including the question about what is important about life, accepting imperfections in oneself, recognising that doctors are people too^46^ and having a desire to now“*enjoy and be grateful for all the possibilities that you get in life, because they can suddenly be taken we from you*. *After all, doctors can also get sick*”.^45^ ^p.6^

### 3.6 Outcome: Disengagement

We identified only one negative outcome in our data, that of Disengagement. Here, the very act of asking students to engage with stories and storytelling led them to turn away from their learning. This was identified in four of the thirteen articles in our study.^33^, ^48^, ^53^, ^54^

CMO #9: “In the context of Engaging with Stories, the mechanism of Resistance leads to the outcome of Disengagement”

Here, students' emotional Resistance to the Narrative Medicine pedagogy (see excerpt in Table 3 the use of *“contrived”*) indicating a level of emotional discomfort with the teaching method, suggesting it felt forced or inauthentic, which could hinder their ability to fully embrace or benefit from it.^53^ Indeed, there is a feeling that the structured method “*cheapens*” the experience, implying a viewpoint that this rigid, structured approach they experienced lacks authenticity and may oversimplify complex human interactions. Additionally, students emotional Resistance in terms of deeply engaging with illness narratives “*indicate discomfort on the part of the student with moving to the point of view of the patient, a potential significant challenge to the development of empathy and patient‐centeredness*”.,^54^ ^p.327^ Indeed, for some this emotional Resistance came about due to the seemingly voyeuristic nature of conversational enquiry into patient's lives, “*I felt kind of bad that she was disclosing … extremely personal information to me, a total stranger, and I'm not directly participating in her healthcare”* which can require strong justification that “*this is for their healthcare*”,^33^ ^p. 12^ for it to become remotely acceptable.

Our findings collectively illustrate how narrative‐based interventions activate distinct mechanisms to produce diverse outcomes, offering valuable insights into their potential to foster holistic care and professional growth while highlighting areas for improvement.

## 4 DISCUSSION

Our study explored how Narrative Medicine influences medical students' preparedness for holistic care, using a realist synthesis approach. Our rigorous CMO analysis identified how the mechanisms of *Deep Observation, Reflection* and *Deeper Patient Engagement* foster holistic patient connections and professional growth. These mechanisms, activated across the contexts of *Storytelling Activities with Patients, Storytelling Skills* and *Engaging with Stories*, contribute to key positive anticipated and unanticipated outcomes including *Enhanced Holistic Patient Connection, Improved Patient Care, Enhanced Ability to Work with Colleagues*, and *Enhanced Self‐Perspective*. However, we also identified the negative outcome of *Avoidance*, which led to *Disengagement*, underscoring the complexity of implementing narrative practices in medical education.

### 4.1 Storytelling in Enhancing Holistic Care

Storytelling was identified as a unifying construct across the CMO configurations, highlighting its pivotal role in fostering holistic care. The three story‐related contexts—Storytelling Activities, Storytelling Skills and Engaging with Stories—offer distinct but complementary pathways for developing relational and reflective competencies. Storytelling Activities allowed students to engage directly with patients, listening to and recounting their narratives, thereby fostering trust and empathy. These interactions bridged technical expertise with humanistic care, equipping students to address patients' multifaceted needs.^29^, ^48^ This aligns with constructivist epistemologies that view illness as a socially and culturally mediated experience, rather than a purely biomedical one.^20^, ^56^ Such a perspective supports an holistic care paradigm that critiques the reductionist biomedical model and advocates for a more integrated, person‐centered understanding of health and illness.

Storytelling Skills emphasise the technical and creative elements of storytelling, such as metaphor, narrative structure and tone.^57^, ^58^, ^59^ By honing these skills, students improved their ability to communicate complex ideas and navigate patient interactions with greater nuance.^16^, ^50^ Meanwhile, Engaging with Stories—the most prominent context—immersed learners in reflective writing, visual arts and narrative photography. These practices deepened their understanding of patients' lived experiences and enhanced their ability to respond to emotional, social and psychological dimensions of care.^52^, ^56^

This layered model offers conceptual clarity to previous reviews, which largely described narrative medicine interventions without articulating how specific storytelling practices generate holistic outcomes.^11^, ^12^, ^15^, ^60^ Our synthesis contributes explanatory depth by mapping storytelling contexts to mechanisms such as Deep Observation and Reflection, which support the development of empathy, trust and cultural responsiveness. This grounding in a realist framework advances current evidence by identifying why narrative practices succeed.

Among the outcomes we identified, Enhanced Holistic Patient Connection was the most significant, demonstrating storytelling's transformative potential in fostering trust and culturally sensitive care.^5^, ^56^ Additionally, the holistic framework advocates for healthcare practices that integrate diverse therapeutic approaches, prioritise patient empowerment and address the root causes of illness.^20^ By recognising patients as whole individuals within their cultural and social contexts, storytelling facilitates more personalised and effective care delivery.

### 4.2 Storytelling in Enhancing Patient Care Outcomes

Storytelling also played a pivotal role in improving patient care outcomes by emphasising both the technical and creative aspects of narrative practices. Through Storytelling Skills, students developed the ability to communicate effectively, developing a deep level of trust with patients.^9^ The mechanism of Deep Observation enabled students to interpret patients' clinical histories with greater nuance, addressing complex needs and integrating emotional insights into clinical decision‐making, thereby bridging the gap between technical expertise and compassionate care.^16^, ^20^ This integration of humanities into medical education further fostered critical thinking and empathy, enriching clinical care by connecting technical proficiency with holistic patient care.^61^

Central to this process was the cultivation of Narrative Competence—the capacity to recognise, interpret and respond to patients' stories—storytelling enhanced students' empathetic engagement and strengthened therapeutic alliances, promoting trust and deeper connections.^27^ These findings align with patient experiences from narrative practice, which show emotional benefits such as hope, empowerment and a stronger connection with clinicians, though some patients also reported distress when recalling traumatic memories.^62^ These mixed outcomes highlight the importance of guided reflection and ethical facilitation when implementing narrative methods in care. This competency also improved students' diagnostic accuracy and relational skills, enabling them to link technical decision‐making with emotional insight.^13^ Such an approach aligns with the principle that healthcare practitioners must address patients as whole individuals, considering their emotional, mental and social dimensions to achieve better outcomes.^50^, ^56^ By integrating storytelling with clinical expertise, students gained a more comprehensive understanding of patient care, demonstrating the transformative potential of narrative practices in fostering meaningful and effective healthcare delivery.

### 4.3 Professional Growth through Narrative Practices

Beyond the anticipated benefits for patients and patient care, our findings reveal a profound and unexpected outcome: Narrative Medicine interventions have a transformative impact on students' interpersonal relationships with peers. This ripple effect of Narrative Medicine interventions has far‐reaching implications for the future of healthcare, promising a more compassionate and cooperative workplace culture in healthcare.

Mechanisms such as reflection and deep observation promote self‐awareness, resilience and cooperation, traits essential for managing the demands of clinical practice.^53^, ^61^ Through narrative writing, students explored personal values and emotions, reinforcing key elements of professional identity such as moral development and reflective capacity.^39^, ^40^ Although some literature links Narrative Medicine with burnout recognition,^12^ studies in our synthesis did not show students becoming more aware of their own burnout. Instead, they demonstrated increased sensitivity to the emotional burdens faced by peers and supervisors.^16^, ^55^

Deep Observation also improved collegial relationships by fostering empathy and mutual respect. Through narrative practices, students were able to see peers and seniors in a different light, developing greater awareness of interpersonal dynamics and the challenges faced by their overworked colleagues suggesting a link to burnout identification in others, if not themselves.^16^, ^55^ Furthermore, narrative sharing provided opportunities for students to construct meaning from experiences, reinforcing their professional identities and relational skills while complementing technical expertise.^56^, ^61^ These mechanisms not only complement technical training but are foundational for shaping compassionate, resilient physicians.^39^, ^40^

### 4.4 Challenges and Areas for Improvement

Despite its transformative potential, the implementation of storytelling practices is not without challenges. Emotional resistance among students, often stemming from perceptions of storytelling exercises as rigid or inauthentic, can hinder engagement.^48^, ^54^ This resistance may reduce the richness of human interactions, limiting empathy development and the ability to connect with patient narratives. Additionally, students' discomfort with exposing vulnerability during reflective practices may impede deeper engagement.^53^, ^63^

This discomfort may lead students to adopt emotional avoidance strategies such as suppression or detachment, particularly in environments lacking psychological safety. This phenomenon is also evident in other studies. Emotional suppression and inaction have been linked to reduced empathy and professional disengagement.^64^ Fear of judgement discourages honest reflection, resulting in shallow engagement with narrative practices.^65^ The hidden curriculum often encourages emotional detachment as a marker of competence, further reinforcing avoidance.^66^ This can help turn potential obstacles into opportunities for growth.

Another challenge lies in the variability of narrative practice outcomes, particularly regarding Peer Learning. Although Peer Learning fosters collaboration and reflective engagement, its impact on holistic care and patient outcomes remains modest.^16^, ^55^ Structured interactions and supportive environments are critical for maximising its potential.^17^ Additionally, culturally sensitive approaches and flexible formats are necessary to integrate humanities‐based practices into medical curricula effectively.^61^

### 4.5 Implications for Medical Education

The findings of this study reinforce the transformative potential of storytelling in medical education. By integrating contexts such as Storytelling Activities and Engaging with Stories, educators can design programmes that develop compassionate, reflective and competent physicians. Supportive learning environments, guided reflection and culturally sensitive practices are essential for fostering resilience, self‐awareness and relational competence.^17^, ^61^, ^63^

Future programmes should aim to address the identified challenges, ensuring that Narrative Medicine are accessible, relevant and adaptable to diverse educational and clinical settings. For example, developing humanistic care through digital platform for storytelling activities might be one way of bringing Narrative Medicine to a larger, inter‐professional student cohort.^13^, ^67^ Additionally, incorporating peer learning and flexible formats into medical curricula could further enhance the integration of storytelling practices, preparing students to deliver empathetic, patient‐centred care that aligns with the holistic ethos of modern healthcare.

### 4.6 Implications for Research

Our research also highlights the need for further work in this area. The fact that we identified only 13 articles, most of which originated from the USA, suggests that a broader international research effort is required. To strengthen the evidence base for Narrative Medicine and its impact on readiness for holistic care, future research should prioritise rigorous and systematic investigations across the Global South as well as investigating a wider geographic spread across the Global North. One crucial step is to conduct longitudinal studies that track the effects of Narrative Medicine on students' preparedness to provide holistic care over time, not only their readiness to practice.^68^, ^69^ This would involve collecting data at multiple time points, from the initial introduction to narrative medicine to long‐term follow‐up interviews.^70^

Another important area for future research is the clinical impact of Narrative Medicine on patient outcomes. Studies show that its integration into practice can improve therapeutic relationships, reduce distress and enhance empathy and communication. Narrative practices have been shown to alleviate physical and emotional distress,^29^ support self‐reflection and symptom relief in cancer patients^71^ and improve empathy and communication during clinical internships.^72^ They have also been used to enhance person‐centered care and therapeutic alliance in oncology^73^ and to improve interdisciplinary understanding in geriatric care.^15^

Although recent studies have begun to explore the clinical benefits of Narrative Medicine, there remains a lack of comprehensive investigations into its long‐term impact on patient care, particularly in relation to clinicians' application of holistic care principles. Further research is needed to clarify the specific components and mechanisms that drive its effectiveness in clinical settings. A sustained and coordinated research agenda is essential to fully elucidate Narrative Medicine's role in preparing clinicians for holistic, patient‐centred care and to strengthen its evidence base across diverse healthcare contexts.

### 4.7 Strengths and Limitations

This study's strengths include its theory‐driven design, adherence to RAMESES standards and systematic data coding using ATLAS.ti, with groundedness analysis enhancing the conceptual depth of the synthesis. The two‐phase search strategy supported both foundational programme theory development and the integration of more recent literature, improving the timeliness and relevance of findings. A key strength lies in the development of nine empirically grounded CMO configurations, which offer explanatory insight into how Narrative Medicine operate within varied educational settings. The analysis distinguishes between storytelling contexts and mechanisms, revealing how specific pedagogical practices activate outcomes such as empathy, professional identity formation and deeper patient engagement. The inclusion of both positive and negative mechanisms, such as emotional resistance leading to disengagement, adds nuance and credibility to the findings.

Nonetheless, there are several limitations to consider. Most included studies originated from the United States, which may limit transferability across cultural and institutional settings. Although a wide range of sources, including non‐English and grey literature, were screened, only peer‐reviewed studies met the final inclusion criteria. As a result, studies from underrepresented regions, particularly the Global South, were not represented in the final synthesis. This may have limited the cultural scope of the findings. These constraints underscore the need for future research that is geographically diverse, methodologically inclusive and longitudinal in design to further refine and test the programme theory.

## AUTHOR CONTRIBUTIONS

CDH, RNA and LVM contributed to the study's conceptualisation, data analysis, synthesis and interpretation. They also played a key role in manuscript writing, reviewing and finalising. LVM and RNA conducted primary data extraction and quality appraisal, managed coding using ATLAS.ti, and refined the CMO configurations. YH conducted the initial study screening and participated in study selection alongside CDH and YSM. YSM contributed to data interpretation and critically revised the manuscript. All authors reviewed and approved the final version of the manuscript.

## CONFLICT OF INTEREST STATEMENT

The authors declare that they have no conflict of interest.

## ETHICS APPROVAL AND CONSENT TO PARTICIPATE

Ethical approval for this study was obtained from the Chang Gung Memorial Hospital and Chang Gung University Institutional Review Board (IRB No. 201601857B0C601).

## CONSENT TO PUBLISH

No individual data is included in this study.

## Supporting information


**Table S1.** Summary of findings of studies reviewed (n = 13).

## Data Availability

The data are kept at the Chang Gung Medical Education Research Center, Chang Gung Memorial Hospital, Chang Gung University College of Medicine, Taipei, Taiwan. Any questions or requests regarding the data can be addressed to Chien‐Da Huang (cdhuang@adm.cgmh.org.tw).

## References

[1] Isaac M . Role of humanities in modern medical education. Curr Opin Psychiatry. 2023;36(5):347‐351. doi:10.1097/YCO.0000000000000884 37458498

[2] Scott PA . The relationship between the arts and medicine. Med Humanit. 2000;26(1):3‐8. doi:10.1136/mh.26.1.3 23669582

[3] Montgomery A , Panagopoulou E , Esmail A , Richards T , Maslach C . Burnout in healthcare: the case for organisational change. BMJ. 2019;366:l4774. doi:10.1136/bmj.l4774 31362957

[4] Szawarski P . Medicine and the human factor. Postgrad Med J. 2020;96(1142):784‐787. doi:10.1136/postgradmedj-2020-138943 33115910

[5] Kerins J , Smith SE , Phillips EC , Clarke B , Hamilton AL , Tallentire VR . Exploring transformative learning when developing medical students' non‐technical skills. Med Educ. 2020;54(3):264‐274. doi:10.1111/medu.14062 31954079

[6] Chen G . The Effective Reduction of Violence Against Doctors Through the Improvement of Medical Humanistic Care. J Multidiscip Healthc. 2023;16:1403‐1407. doi:10.2147/JMDH.S411674 37228867 PMC10202701

[7] Engel GL . From biomedical to biopsychosocial: being scientific in the human domain. Psychosomatics. 1997;38(6):521‐528. doi:10.1016/S0033-3182(97)71396-3 9427848

[8] Borrell‐Carrió F , Suchman AL , Epstein RM . The biopsychosocial model 25 years later: principles, practice, and scientific inquiry. Ann Fam Med. 2004;2(6):576‐582. doi:10.1370/afm.245 15576544 PMC1466742

[9] Charon R . Narrative medicine a model for empathy, reflection, profession, and trust. JAMA. 2001;286(15):1897‐1902. doi:10.1001/jama.286.15.1897 11597295

[10] Ajjawi R , Rees C , Monrouxe L . Learning clinical skills during bedside teaching: a video observation study drawing on insights from activity theory. Med Educ. 2014;48:128‐129.

[11] Milota MM , van Thiel GJMW , van Delden JJM . Narrative medicine as a medical education tool: A systematic review. Med Teach. 2019;41(7):802‐810. doi:10.1080/0142159X.2019.1584274 30983460

[12] Remein CD , Childs E , Pasco JC , et al. Content and outcomes of narrative medicine programmes: a systematic review of the literature through 2019. BMJ Open. 2020;10. 10.1136/bmjopen-2019-031568 PMC7045204 31988222

[13] Efthymiou E . Integrating digital and narrative medicine in modern healthcare: a systematic review. Med Educ Online. 2025;30(1):2475979. doi:10.1080/10872981.2025.2475979 40327849 PMC12057780

[14] Kuo P‐Y , Chu S‐Y , Lin M‐J , Tseng T‐C . Narrative medicine in pediatric medical education and patient care: A scoping review. Tzu Chi Med J. 2025;37(2):167‐174. doi:10.4103/tcmj.tcmj_181_24 40321965 PMC12048120

[15] Paul TK , Reddy Y , Gnanakumar A , et al. Narrative medicine interventions for oncology clinicians: a systematic review. Support Care Cancer. 2024;32(4):241. doi:10.1007/s00520-024-08434-1 38512594 PMC11439222

[16] Arntfield SL , Slesar K , Dickson J , Charon R . Narrative medicine as a means of training medical students toward residency competencies. Patient Educ Couns. 2013;91(3):280‐286. doi:10.1016/j.pec.2013.01.014 23462070 PMC3992707

[17] Liao K‐C , Peng C‐H , Snell L , Wang X , Huang C‐D , Saroyan A . Understanding the lived experiences of medical learners in a narrative medicine course: a phenomenological study. BMC Med Educ. 2021;21(1):321. doi:10.1186/s12909-021-02741-5 34090423 PMC8180022

[18] Wesley T , Hamer D , Karam G . Implementing a narrative medicine curriculum during the internship year: an internal medicine residency program experience. Perm J. 2018;22:17‐187. doi:10.7812/TPP/17-187 PMC5922967 29702059

[19] Acosta LMY , Ely EW . Holistic care in healthy aging: caring for the wholly and holy human. Aging Cell. 2024;23(1):e14021. doi:10.1111/acel.14021 37873723 PMC10776114

[20] Gordon JS . The Paradigm of Holistic Medicine. Routledge; 2018.

[21] Wender R . Integrating oncology, psychosocial, and medical care: The path forward. Psychooncology. 2020;29(3):461‐464. doi:10.1002/pon.5322 31876041

[22] Olson AW , Stratton TP , Isetts BJ , Vaidyanathan R , Van Hooser CJ , Schommer JC . Seeing the elephant: a systematic scoping review and comparison of patient‐centeredness conceptualizations from three seminal perspectives. J Multidiscip Healthc. 2021;973‐986. 33953566 10.2147/JMDH.S299765 PMC8092624

[23] van der Veen S , Evans N , Huisman M , Welch Saleeby P , Widdershoven G . Toward a paradigm shift in healthcare: using the International Classification of Functioning, Disability and Health (ICF) and the capability approach (CA) jointly in theory and practice. Disabil Rehabil. 2023;45(14):2382‐2389. doi:10.1080/09638288.2022.2089737 35732595

[24] Barkley L , Taliaferro LA , Baker K , Garcia J . The holistic athletic healthcare model: addressing the developmental, social, and cultural needs of collegiate athletes. J High Educ Athl Innov. 2018;1(3):26‐47.

[25] Young J , Cund A , Renshaw M , Quigley A , Snowden A . Improving the care of cancer patients: holistic needs assessment. Br J Nurs. 2015;24(Sup4):S17‐S20. doi:10.12968/bjon.2015.24.Sup4.S17 25723367

[26] Charon R . Narrative Medicine: Honoring the Illness. Oxford University Press; 2006.

[27] Leopold SS . Editorial: What is narrative medicine, and why should we use it in orthopaedic practice? Clin Orthop Relat Res. 2018;476(11):2105‐2107. doi:10.1097/CORR.0000000000000504 30239354 PMC6259984

[28] Quah ELY , Chua KZY , Lin CKR , et al. The role of patients' stories in medicine: a systematic scoping review. BMC Palliat Care. 2023;22(1):199. doi:10.1186/s12904-023-01319-w 38087237 PMC10714554

[29] Fioretti C , Mazzocco K , Riva S , Oliveri S , Masiero M , Pravettoni G . Research studies on patients' illness experience using the narrative medicine approach: a systematic review. BMJ Open. 2016;6(7):e011220. 10.1136/bmjopen-2016-011220 PMC4947803 27417197

[30] Amatori G , Buccolo M . Narrative Medicine Practices: Emotional Literacy and Inclusion Through Reading. In: Cortijo Ocaña A , Peres VM , Orazi V , eds. Improving Mental Health and Wellbeing Through Bibliotherapy. IGI Global; 2024:163‐187.

[31] Xiao S , Yuan J , Lan H , et al. Investigation of clinical medicine undergraduates' recognition of narrative medicine. BMC Med Educ. 2024;24(1):321. doi:10.1186/s12909-024-05279-4 38515120 PMC10958904

[32] Huang C‐D , Liao K‐C , Chung F‐T , et al. Different perceptions of narrative medicine between Western and Chinese medicine students. BMC Med Educ. 2017;17(1):85. doi:10.1186/s12909-017-0925-0 28490362 PMC5424351

[33] Morgan S , Young M , Demers L , Pasco JC , Jindal S . Combating ageism in medical education with narrative medicine. Gerontol Geriatr Educ. 2025;46(1):5‐16. doi:10.1080/02701960.2024.2302594 38217514

[34] Pawson R , Greenhalgh T , Harvey G , Walshe K . Realist review – a new method of systematic review designed for complex policy interventions. J Health Serv Res Policy. 2005;10(Suppl 1):21‐34. doi:10.1258/1355819054308530 16053581

[35] Wong G , Greenhalgh T , Westhorp G , Buckingham J , Pawson R . Rameses publication standards: realist syntheses. BMC Med. 2013;11(1):1‐14. 23360677 10.1186/1741-7015-11-21 PMC3558331

[36] Greenhalgh, T , Pawson, R , Wong, G , et al Realist evaluation, realist synthesis, realist research – what's in a name? [The RAMESES II Project]. In: NIHR, editor. 2017.

[37] Rees CE , Crampton PES , Nguyen VNB , Monrouxe LV . Introducing realist approaches in health professions education research. In: Rees CE , Monrouxe LV , O'Brien BC , Gordon LJ , Palermo C , eds. Foundations of health professions education research: Principles, perspectives, and practices. John Wiley & Sons; 2023.

[38] Huang Y , Monrouxe LV , Huang CD . The influence of narrative medicine on medical students' readiness for holistic care practice: a realist synthesis protocol. BMJ Open. 2019;9(8):e029588. doi:10.1136/bmjopen-2019-029588 PMC6687057 31377710

[39] Cruess RL , Cruess SR , Boudreau JD , Snell L , Steinert Y . A schematic representation of the professional identity formation and socialization of medical students and residents: a guide for medical educators. Acad Med. 2015;90(6):718‐725. doi:10.1097/ACM.0000000000000700 25785682

[40] Wald HS . Professional identity (trans)formation in medical education: reflection, relationship, resilience. Acad Med. 2015;90(6):701‐706. doi:10.1097/ACM.0000000000000731 25881651

[41] Matthew JP , Joanne EM , Patrick MB , et al. The PRISMA 2020 statement: an updated guideline for reporting systematic reviews. BMJ. 2021;372:n71. 33782057 10.1136/bmj.n71 PMC8005924

[42] Brennan N , Bryce M , Pearson M , Wong G , Cooper C , Archer J . Understanding how appraisal of doctors produces its effects: a realist review protocol. BMJ Open. 2014;4(6):e005466. doi:10.1136/bmjopen-2014-005466 PMC4067866 24958211

[43] Friese S . Qualitative Data Analysis with ATLAS.ti. 55 City Road. SAGE Publications Ltd; 2012. https://methods.sagepub.com/book/mono/qualitative-data-analysis-with-atlas/toc

[44] Booth, A , Wright, J , Briscoe, S . Scoping and searching to support realist approaches. Doing realist research London: Sage. 2018:147–166.

[45] Leijenaar E , Eijkelboom C , Milota M . “An invitation to think differently”: a narrative medicine intervention using books and films to stimulate medical students' reflection and patient‐centeredness. BMC Med Educ. 2023;23(1):568. doi:10.1186/s12909-023-04492-x 37563708 PMC10416442

[46] Stumbar SE , Phan M , Samuels M . An exploratory study of a fourth‐year narrative medicine elective: promoting strategies for personal well‐being and improved patient care. South Med J. 2023;116(1):42‐45. doi:10.14423/SMJ.0000000000001497 36578117

[47] Huang CD , Jenq CC , Liao KC , Lii SC , Huang CH , Wang TY . How does narrative medicine impact medical trainees' learning of professionalism? A qualitative study. BMC Med Educ. 2021;21(1):391. doi:10.1186/s12909-021-02823-4 34289848 PMC8296619

[48] Chretien KC , Swenson R , Yoon B , et al. Tell Me Your Story: A Pilot Narrative Medicine Curriculum During the Medicine Clerkship. J Gen Intern Med. 2015;30(7):1025‐1028. doi:10.1007/s11606-015-3211-z 25670397 PMC4471011

[49] DasGupta S , Charon R . Personal illness narratives: Using reflective writing to teach empathy. Acad Med. 2004;79(4):351‐356. 15044169 10.1097/00001888-200404000-00013

[50] Garrison D , Lyness JM , Frank JB , Epstein RM . Qualitative analysis of medical student impressions of a narrative exercise in the third‐year psychiatry clerkship. Acad Med. 2011;86(1):85‐89. doi:10.1097/ACM.0b013e3181ff7a63 21099398

[51] Green MJ . Comics and medicine: peering into the process of professional identity formation. Acad Med. 2015;90(6):774‐779. doi:10.1097/ACM.0000000000000703 25853686

[52] Lemay M , Encandela J , Sanders L , Reisman A . Writing Well: The Long‐Term Effect on Empathy, Observation, and Physician Writing Through a Residency Writers' Workshop. J Grad Med Educ. 2017;9(3):357‐360. doi:10.4300/JGME-D-16-00366.1 28638517 PMC5476388

[53] Miller E , Balmer D , Hermann N , Graham G , Charon R . Sounding narrative medicine: studying students' professional identity development at Columbia University College of Physicians and Surgeons. Acad Med. 2014;89(2):335‐342. doi:10.1097/ACM.0000000000000098 24362390 PMC4002760

[54] Brown R , Griggs M , Cummins J , Nittler J , Gordy‐Panhorst K , Hoffman KG . What can a brief narrative exercise reveal about medical students' development as patient‐centered physicians and their attitudes toward patients with mental illness? Acad Psychiatry. 2015;39(3):324‐328. doi:10.1007/s40596-015-0291-3 25835458

[55] Levine RB , Kern DE , Wright SM . The impact of prompted narrative writing during internship on reflective practice: a qualitative study. Adv Health Sci Educ Theory Pract. 2008;13(5):723‐733. doi:10.1007/s10459-007-9079-x 17899421

[56] Warmington SG . Storytelling Encounters as Medical Education: Crafting Relational Identity. Taylor & Francis Group; 2020.

[57] Mitchell K , Skirton H , Monrouxe L . Amelioration, regeneration, acquiescent and discordant: an exploration of narrative types and metaphor use in people with aphasia. Dis Soc. 2011;26(3):321‐335. doi:10.1080/09687599.2011.560415

[58] Rees C , Knight L , Cleland J . Medical educators' metaphoric talk about their assessment relationships with students: “You don't want to sort of be the one who sticks the knife in them.”. Assess Eval High Educ. 2008;34(4):455‐467.

[59] Rees C , Knight L , Wilkinson C . “Doctors being up there and we being down here”: a metaphorical analysis of talk about doctors, patients and medical students. Soc Sci Med. 2007;65(4):725‐737. 17493722 10.1016/j.socscimed.2007.03.044

[60] Palla I , Turchetti G , Polvani S . Narrative Medicine: Theory, clinical practice and education ‐ a scoping review. BMC Health Serv Res. 2024;24(1):1116. doi:10.1186/s12913-024-11530-x 39334149 PMC11428871

[61] Chiavaroli, N , Huang, C‐D , Monrouxe, L . Learning Medicine With, From, and Through the Humanities. Understanding Medical Education 2018. p. 223–237.

[62] Yang Y , Xu J , Hu Y , Hu J , Jiang A . The experience of patients with cancer on narrative practice: A systematic review and meta‐synthesis. Health Expect. 2020;23(2):274‐283. doi:10.1111/hex.13003 31944492 PMC7104641

[63] Pattanaik D , Purvis E , Jeffrey D . Storytelling: A learning tool to enhance medical students' empathy, attentive listening, clinical curiosity and reflection. J R Coll Physicians Edinb. 2024;54(4):325‐329. doi:10.1177/14782715241299839 39539201

[64] Doulougeri K , Panagopoulou E , Montgomery A . (How) do medical students regulate their emotions? BMC Med Educ. 2016;16(1):312. doi:10.1186/s12909-016-0832-9 27955653 PMC5154027

[65] Gathu C . Facilitators and barriers of reflective learning in postgraduate medical education: a narrative review. J Med Educat Curri Develop. 2022;9:23821205221096106. doi:10.1177/23821205221096106 PMC9069595 35529178

[66] Ní Mhurchú M , Cantillon P . Reflective practice in medicine: The hidden curriculum challenge. Clin Teach. 2024;21(2):e13682. doi:10.1111/tct.13682 37855062

[67] Deng X , Ye M , Li W , et al. Development of a humanistic care digital storytelling programme for intensive care unit nursing students: feasibility and satisfaction analysis. Nurse Educ Today. 2024;132:105998. doi:10.1016/j.nedt.2023.105998 37939571

[68] Monrouxe LV , Bullock A , Gormley G , et al. New graduate doctors' preparedness for practice: a multistakeholder, multicentre narrative study. BMJ Open. 2018;8(8):e023146. doi:10.1136/bmjopen-2018-023146 PMC6119440 30158236

[69] Ottrey E , Rees CE , Kemp C , et al. Exploring preparedness transitions in medicine and pharmacy: a qualitative longitudinal study to inform multiprofessional learning opportunities. Adv Health Sci Educ Theory Pract. 2024;30(3):1‐24. doi:10.1007/s10459-024-10372-w PMC12119723 39285010

[70] Monrouxe, LV , Brown, ME , Ottrey, E , Gordon, LJ . Introducing interpretivist approaches in health professions education research. Foundations of health professions education research: principles, perspectives and practices 2023:122–144.

[71] Makoff EL , Rehman R , Ming L , Iqbal S . Can narrative medicine methods improve well‐being in patients with gastrointestinal malignancy? J Clin Oncol. 2024;42(16_suppl):e24091.

[72] Yuan J , Xiangyang Z , Yan C , Hua L , Ke C , Xiao S . Narrative medicine in clinical internship teaching practice. Med Educ Online. 2023;28(1):2258000. 37722672 10.1080/10872981.2023.2258000 PMC10512813

[73] Cercato MC , Onesti CE , Vari S , et al. Narrative medicine: a digital diary in the management of patients with bone and soft tissue sarcoma—a multidisciplinary pilot study. J Clin Med. 2023;12(23):7218. doi:10.3390/jcm12237218 38068266 PMC10706943

