# Supplementary material for: The influence of narrative medicine on medical students' readiness for holistic care practice: A realist synthesis
Source: Med Educ. 2025 Aug 22;60(3):230–46. doi: 10.1111/medu.70024 (PMC12913228; doi:10.1111/medu.70024)
Supplement: Supplementary file 1 — Table S1. Summary of findings of studies reviewed (n = 13). [file MEDU-60-230-s001.docx]

# Supplementary File

TABLE OF CONTENT

[**Supl. 1: Search Strategy** 2](#_Toc204254799)

[Phase 1 2](#_Toc204254800)

[Phase 2 4](#_Toc204254801)

[MEDLINE 115 4](#_Toc204254802)

[Scopus 111 4](#_Toc204254803)

[Web of Science 55 5](#_Toc204254804)

[Embase 141 5](#_Toc204254805)

[PRISMA Summary Table 7](#_Toc204254806)

[**Supl. 2: RAMESES** 8](#_Toc204254807)

[**Supl. 3: Summary of Findings from the Reviewed Studies** 10](#_Toc204254808)

# Supl. 1: Search Strategy

## Phase 1

1. TOPIC: (“medical student*”)

2. TOPIC: (“intern”)

3. TOPIC: (“clerk*”)

4. TOPIC: (“residen*”)

5. TOPIC: (“postgraduate trainee*”)

6. TOPIC: (“postgraduate year”)

7. TOPIC: OR/ 1-6

8. TOPIC: (“patient-center*”)

9. TOPIC: (“patient-centre*”)

10. TOPIC: (“person-center*”)

11. TOPIC: (“person-centre*”)

12. TOPIC: (“holistic health”)

13. TOPIC: (“holistic medicine”)

14. TOPIC: (“holistic car*”)

15. TOPIC: (“holistic practic*”)

16. TOPIC: (“biopsychosocial”)

17. TOPIC: (“holistic need”)

18. TOPIC: (“whole-patient car*”)

19. TOPIC: (“total-patient car*”)

20. TOPIC: (holistic AND “medical educat*”)

21. TOPIC: (“empath*”)

22. TOPIC: (“compassion*”)

23. TOPIC: (“sensitiv*”)

24. TOPIC: (“social*”)

25. TOPIC: (“listener”)

26. TOPIC: (“individual*”)

27. TOPIC: (“respect*”)

28. TOPIC: (“empower*”)

29. TOPIC: (“communicat*”)

30. TOPIC: (“reflect*”)

31. TOPIC: OR/ 8-30

32. TOPIC: (“narrative medicine”)

33. TOPIC: (“narrative medical”)

34. TOPIC: (“narrative train*”)

35. TOPIC: (“narrative medicine train*”)

36. TOPIC: (narrative AND “medical educat*”)

37. TOPIC: OR/ 32-36

38. TOPIC: 7 AND 31 AND 37

39. Limit 39 to (yr= “2008-2018”)

40. Limit 39 to language English and Mandarin

## Phase 2

### MEDLINE 115

| Set# | Searched for | Databases | Results |
| --- | --- | --- | --- |
| S1 | ((medical student) OR intern* OR residen* OR (postgraduate trainee*) OR (postgraduate year)) AND la.exact("English" OR "Chinese") | MEDLINE® | 1398452 |
| S2 | (patient-center* OR patient-centre* OR person-center* OR person-centre* OR (holistic health) OR (holistic medicine) OR (holistic car*) OR (holistic practic*) OR biopsychosocial OR (holistic need*)) AND la.exact("English" OR "Chinese") | MEDLINE® | 120299 |
| S3 | ((narrative medicine) OR (narrative medical) OR (narrative train*) OR (narrative medicine train*)) AND la.exact("English" OR "Chinese") | MEDLINE® | 35742 |
| S4 | ((medical educat*) OR (clinical educat*) OR (health professional educat*)) AND la.exact("English" OR "Chinese") | MEDLINE® | 642147 |
| S5 | ((medical educat*) OR (clinical educat*) OR (health professional educat*)) AND la.exact("English" OR "Chinese") AND pd(20180101-20250630) | MEDLINE® | 247073 |
| S6 | ((medical educat*) OR (clinical educat*) OR (health professional educat*)) AND la.exact("English" OR "Chinese") AND pd(20180101-20250630) | MEDLINE® | 247073 |
| S11 | ([S1] AND [S2] AND [S3] AND [S4]) AND pd(20181001-20250630) | MEDLINE®  These databases are searched for part of your query. | 115 |

### Scopus 111

( TITLE-ABS-KEY ( "medical student" OR "medical students" OR intern OR clerk OR clerks OR resident OR residents OR "postgraduate trainee" OR "postgraduate trainees" OR "postgraduate year" ) ) AND ( TITLE-ABS-KEY ( "patient centered" OR "patient centred" OR "person centered" OR "person centred" OR "holistic health" OR "holistic medicine" OR "holistic care" OR "holistic practice" OR biopsychosocial OR "holistic needs" OR "whole patient care" OR "total patient care" OR ( holistic W/3 "medical education" ) OR empath* OR compassion* OR sensitiv* OR social* OR listener OR individual* OR respect* OR empower* OR communicat* OR reflect* ) ) AND ( TITLE-ABS-KEY ( "narrative medicine" OR "narrative medical" OR "narrative training" OR "narrative medicine training" OR ( narrative W/3 "medical education" ) ) ) AND ( TITLE-ABS-KEY ( "medical education" OR "clinical education" OR "health professional education" ) ) AND PUBYEAR > 2018 AND PUBYEAR < 2025 AND ( LANGUAGE ( english ) OR LANGUAGE ( chinese ) )

Result: 111

### Web of Science 55

(((ALL=("medical student*" OR intern* OR clerk* OR resident* OR "postgraduate trainee*" OR "postgraduate year")) AND ALL=("patient centered" OR "patient centred" OR "person centered" OR "person centred" OR "holistic health" OR "holistic medicine" OR "holistic care" OR "holistic practice" OR biopsychosocial OR "holistic needs" OR "whole patient care" OR "total patient care" OR empathy OR compassion* OR sensitiv* OR social* OR listener OR individual* OR respect* OR empower* OR communicat* OR reflect* OR "holistic medical education")) AND ALL=("narrative medicine" OR "narrative medical" OR "narrative training" OR "narrative medicine training" OR "narrative medical education")) AND ALL=("medical education" OR "clinical education" OR "health professional education")

<https://www.webofscience.com/wos/woscc/summary/5f898004-3e88-4df2-967a-8118b06f8009-016fd7b6b1/relevance/1>

### Embase 141

| **No.** | **Query** | **Results** |
| --- | --- | --- |
| #42 | #7 AND #31 AND #37 AND #41 AND ([chinese]/lim OR [english]/lim) AND [2018-2025]/py | 141 |
| #41 | #38 OR #39 OR #40 | 448861 |
| #40 | 'health professional educat*' | 1262 |
| #39 | 'clinical educat*' | 27574 |
| #38 | 'medical educat*' | 428824 |
| #37 | #32 OR #33 OR #34 OR #35 OR #36 | 1414 |
| #36 | 'narrative adj3 medical educat*' | 0 |
| #35 | 'narrative medicine train*' | 9 |
| #34 | 'narrative train*' | 15 |
| #33 | 'narrative medical' | 60 |
| #32 | 'narrative medicine' | 1353 |
| #31 | #8 OR #9 OR #10 OR #11 OR #12 OR #13 OR #14 OR #15 OR #16 OR #17 OR #18 OR #19 OR #20 OR #21 OR #22 OR #23 OR #24 OR #25 OR #26 OR #27 OR #28 OR #29 OR #30 | 12908863 |
| #30 | 'reflect*' | 1027105 |
| #29 | 'communicat*' | 1381835 |
| #28 | 'empower*' | 74955 |
| #27 | 'respect*' | 5032317 |
| #26 | 'individual*' | 3044442 |
| #25 | 'listener' | 4939 |
| #24 | 'social*' | 2032111 |
| #23 | 'sensitiv*' | 2918166 |
| #22 | 'compassion*' | 31203 |
| #21 | 'empath*' | 51119 |
| #20 | 'holistic adj3 medical educat*' | 0 |
| #19 | 'total patient care' | 280 |
| #18 | 'whole patient care' | 46 |
| #17 | 'holistic need*' | 628 |
| #16 | 'biopsychosocial' | 18790 |
| #15 | 'holistic practice' | 152 |
| #14 | 'holistic care' | 9444 |
| #13 | 'holistic medicine' | 809 |
| #12 | 'holistic health' | 1789 |
| #11 | 'person-centre*' | 8941 |
| #10 | 'person-center*' | 13048 |
| #9 | 'patient-centre*' | 16678 |
| #8 | 'patient-center*' | 48692 |
| #7 | #1 OR #2 OR #3 OR #4 OR #5 OR #6 | 7590385 |
| #6 | postgraduate AND year | 27534 |
| #5 | postgraduate AND trainee* | 6378 |
| #4 | residen* | 566245 |
| #3 | clerk* | 13674 |
| #2 | intern* | 7052604 |
| #1 | 'medical student*' | 129338 |

## PRISMA Summary Table

| **Stage** | **Count** | **Notes** |
| --- | --- | --- |
| Records identified through database searching (Phase 1: 2008–2018) | 1,039 |  |
| Records identified through database searching (Phase 2: 2018–2025) | 422 |  |
| Additional records identified through other sources | 2 | Manual additions |
| **Total records before deduplication** | **1,463** |  |
| Records after duplicates removed | 862 |  |
| Records screened (title/abstract) | 862 |  |
| Records excluded (title/abstract level) | 745 |  |
| **Full-text articles assessed for eligibility** | **117** | 103 from Phase 1, 14 from Phase 2 |
| Full-text articles excluded | 104 | 93 from Phase 1, 11from Phase 2 |
| – Coded but excluded from synthesis (not sufficiently grounded) | 1 | From Phase 2 |
| – Reviewed but not used further | 10 | Not coded, not cited |
| **Studies included in realist synthesis** | **13** | 10 from Phase 1, 3 from Phase 2 |

| **Exclusion Reason** | **Phase 1** | **Phase 2** | **Total (n)** |
| --- | --- | --- | --- |
| Irrelevant papers | 48 | 0 | 46 |
| Descriptive articles (lacking explanatory depth) | 19 | 0 | 19 |
| Conference abstracts | 23 | 0 | 23 |
| Pilot study | 1 | 0 | 1 |
| Non-English/Mandarin articles | 2 | 0 | 2 |
| Conceptually insufficient (not coded or cited in discussion) | 0 | 10 | 10 |
| Limited CMO contribution (coded but not retained) | 0 | 1 | 4 |
| **Total** | **93** | **11** | **104** |

# Supl. 2: RAMESES

List of items to be included when reporting a realist synthesis^1^

| **TITLE** | | | Page |
| --- | --- | --- | --- |
| 1 |  | In the title, identify the document as a realist synthesis or review | P1 |
| **ABSTRACT** | | |  |
| 2 |  | While acknowledging publication requirements and house style, abstracts should ideally contain brief details of: the study's background, review question or objectives; search strategy; methods of selection, appraisal, analysis and synthesis of sources; main results; and implications for practice. | P1-2 |
| **INTRODUCTION** | | |  |
| 3 | Rationale for review | Explain why the review is needed and what it is likely to contribute to existing understanding of the topic area. | P3-5 |
| 4 | Objectives and focus of review | State the objective(s) of the review and/or the review question(s). Define and provide a rationale for the focus of the review. | P6 L1-8 |
| **METHODS** | | |  |
| 5 | Changes in the review process | Any changes made to the review process that was initially planned should be briefly described and justified. | P8 L30-31  P9 L10-14  P10 L16-18 |
| 6 | Rationale for using realist synthesis | Explain why realist synthesis was considered the most appropriate method to use. | P6 L9 – P7 L40 |
| 7 | Scoping the literature | Describe and justify the initial process of exploratory scoping of the literature. | Stage1  P7 L28- P8 L22 |
| 8 | Searching processes | While considering specific requirements of the journal or other publication outlet, state and provide a rationale for how the iterative searching was done. Provide details on all the sources accessed for information in the review. Where searching in electronic databases has taken place, the details should include, for example, name of database, search terms, dates of coverage and date last searched. If individuals familiar with the relevant literature and/or topic area were contacted, indicate how they were identified and selected. | Stage 2 P8  Supplementary 1 |
| 9 | Selection and appraisal of documents | Explain how judgements were made about including and excluding data from documents, and justify these. | Stage 3 P9  Figure 1 |
| 10 | Data extraction | Describe and explain which data or information were extracted from the included documents and justify this selection. | Stage 4  P9 |
| 11 | Analysis and synthesis processes | Describe the analysis and synthesis processes in detail. This section should include information on the constructs analyzed and describe the analytic process. | Stage 5  P10 |
| **RESULTS** | | |  |
| 12 | Document flow diagram | Provide details on the number of documents assessed for eligibility and included in the review with reasons for exclusion at each stage as well as an indication of their source of origin (for example, from searching databases, reference lists and so on). You may consider using the example templates (which are likely to need modification to suit the data) that are provided. | Figure 2 PRISMA |
| 13 | Document characteristics | Provide information on the characteristics of the documents included in the review. | Supplementary 3 |
| 14 | Main findings | Present the key findings with a specific focus on theory building and testing. | 11-17 |
| **DISCUSSION** | | |  |
| 15 | Summary of findings | Summarize the main findings, taking into account the review's objective(s), research question(s), focus and intended audience(s). | P17 L10-12 |
| 16 | Strengths, limitations and future research directions | Discuss both the strengths of the review and its limitations. These should include (but need not be restricted to) (a) consideration of all the steps in the review process and (b) comment on the overall strength of evidence supporting the explanatory insights which emerged. The limitations identified may point to areas where further work is needed. | 4.7 P22 L17 |
| 17 | Comparison with existing literature | Where applicable, compare and contrast the review's findings with the existing literature (for example, other reviews) on the same topic. | P18 L14-16  P19 L10-18  P20 L2  P20 L23-30 |
| 18 | Conclusion and recommendations | List the main implications of the findings and place these in the context of other relevant literature. If appropriate, offer recommendations for policy and practice. | P21 L7 – P22 L16 |
| 19 | Funding | Provide details of funding source (if any) for the review, the role played by the funder (if any) and any conflicts of interests of the reviewers. | 24 |

# Supl. 3: Summary of Findings from the Reviewed Studies

Table 1 Summary of findings of studies reviewed (n=13)

| **No.** | **Author** | **Type of study** | **Intervention type** | **Study location** | **Participants** | **Total participants** | **Settings/ Location** | **Sites** | **Intended**  **Outcomes (+)** | **Unintended Outcomes (-)** |
| --- | --- | --- | --- | --- | --- | --- | --- | --- | --- | --- |
| PHASE１ | | | | | | | | | | |
|  | Arntfield et al, 2013 ^2^ | Qualitative (grounded theory) study | Required courses:   - Reading contemporary fiction novel - Discussion of assigned readings - Fiction writing workshop - Reflections on critical junctures | USA | Fourth year medical student | 12 | Medical school | Single Site | Increased motivation to learn and practice Narrative Medicine, professional growth, increased self-awareness, and increased Empathy | Narrative Medicine activities was perceived as distractions from the essential biomedicine course |
|  | Brown et al, 2015^3^ | Qualitative (descriptive) study | Required course:   - Narrative exercise after students interviewing the patients | USA | First and Single site second year | 193 | Medical school | Single site | The activity promotes self-reflection, encourages students to understand patients’ perspectives, and provides insight of the goal of patient-centeredness. | Students had varied small group experiences due to inconsistent facilitator guidance, and some were offended by the term "fantasies" in the prompt, feeling it undermined the legitimacy of the patient's perspective. |
|  | Chretien et al, 2015^4^ | Qualitative (descriptive) study | Seminar:   - Introductory long-hour session - Story-telling - Group reflection | USA | Clerk | 31 | Combination | Single site | The Narrative Medicine activities promotes a good relationship development with patients, improved enhancement of patient care, increased empathy, and improvement in knowledge | The patient’s response is considered to deviate from expectations, also lack of time to implement Narrative Medicine activities in the clinical practice |
|  | DasGupta and Charon, 2004 ^5^ | Qualitative (descriptive) study | Seminar:   - Narrative writing exercise | USA | Medical Student Second year | 16 | Medical school | Single site | Seminar well received by participants, help them to clarify their issues, enhanced empathy and explore their perspective and biases, growing confidence, | Challenging emotion and uncomfortable feeling |
|  | Garrison et al, 2011^6^ | Qualitative (descriptive) study | Seminar:   - Introduction to the narrative approaches - Interaction between students and patients - Writing narrative essay - Discussion | USA | Clerk | 46 | Medical school | Single site | Promotes validating patients' perspectives, increased connection and therapeutic alliance with their patients, relationship building, and enhancement in patient care | The written narrative lacked the jargon and details that a thorough patients’ medical history. |
|  | Green, 2015^7^ | Mix method (qualitative content analysis and likert scale) | Seminar:   - Reading graphic narratives - Sharing in comic format | USA | Fourth year | 42 | Medical school | Single site | Improved better communication, increased self-development, and promotes patient-centeredness | N/A (Not mentioned in the article) |
|  | Lemay et al, 2017^8^ | Mixed method | Workshop:   - Creative writing | USA | resident | 130- | Medical school | Single site | Promotes self-reflection, empathy, and self-care from writing and discussing narrative experiences, and showed improvement in knowledge | N/A (Not mentioned in the article) |
|  | Levine et al, 2008^9^ | Qualitative (descriptive) study | Residency program:   - Seminar - Reflective writing | USA | Interns | 32 | Combination (community-based, university affiliated) | Multiple sites (9 different internal medicine residency sites) | Improved self-reflection, promotes self-development (motivation to improve personally and professionally), and enhanced self-care | N/A (Not mentioned in the article) |
|  | Miller, 2014^10^ | Qualitative (descriptive) study | Seminar :   - Close reading/writing - Visual art - Social sciences | USA | Medical student | 130 | Medical school | Single site | Improved connection with patients, peers, colleagues, and communities (build a good relationship), improved knowledge and enhance patient care | Feeling stressful with the tasks, indicating disagreement with learning activity, and showed motivation to change the curriculum |
|  | Wesley et al, 2018^11^ | Qualitative (descriptive) study | Seminar:   - Conferences - Reflective sessions | USA | Interns | 13 | Hospital | Single site | enhance patient care, promotes self-care and self-development, and showed outcomes on the physician wellness | N/A (Not mentioned in the article) |
| **PHASE 2** | | | | | | | | | | |
|  | Morgan et al. (2025)^12^ | Mixed-methods | Narrative medicine (My Life, My Story) | USA | Fourth-year medical students, faculty | 151 | Hospital | Single Site | Improved student attitudes toward older adults; reduced ageism; increased awareness of social determinants of health, patient humanity, and active societal contributions | Some student discomfort discussing deeply personal content with patients; possible bias from broader Geriatrics rotation context |
|  | Leijenaar et al. (2023)^13^ | Mixed-methods | Narrative medicine using book and film-based reflection | Netherlands | Fourth-year medical students | 345 | Medical school | Single site | Improved patient-centered attitudes; enhanced self-awareness and reflection; appreciation of patient uniqueness; development of communication intentions | Some superficial reflections due to short format; variation in teacher instruction; limited transferability to clinical settings noted |
|  | Stumbar et al. (2023)^14^ | Exploratory mixed-methods | Narrative medicine elective (writing, reading, discussion) | USA | Fourth-year medical students | 17 | Medical school | Single site | Enhanced self-reflection and resilience; improved empathy, patient advocacy, communication, and emotional processing | Small self-selected sample; lack of long-term outcome data; uncertainty about real-world transfer into clinical care |

1. Wong G, Greenhalgh T, Westhorp G, Buckingham J, Pawson R. RAMESES publication standards: realist syntheses. *BMC medicine*. 2013;**11**:1-14.

2. Arntfield SL, Slesar K, Dickson J, Charon R. Narrative medicine as a means of training medical students toward residency competencies. *Patient education and counseling*. 2013;**91**:280-286.

3. Brown R, Griggs M, Cummins J, Nittler J, Gordy-Panhorst K, Hoffman KG. What can a brief narrative exercise reveal about medical students' development as patient-centered physicians and their attitudes toward patients with mental illness? *Academic psychiatry : the journal of the American Association of Directors of Psychiatric Residency Training and the Association for Academic Psychiatry*. 2015;**39**:324-328.

4. Chretien KC, Swenson R, Yoon B, et al. Tell Me Your Story: A Pilot Narrative Medicine Curriculum During the Medicine Clerkship. *Journal of General Internal Medicine*. 2015;**30**:1025-1028.

5. DasGupta S, Charon R. Personal illness narratives: Using reflective writing to teach empathy. *Academic Medicine*. 2004;**79**:351-356.

6. Garrison D, Lyness JM, Frank JB, Epstein RM. Qualitative analysis of medical student impressions of a narrative exercise in the third-year psychiatry clerkship. *Academic Medicine*. 2011;**86**:85-89.

7. Green MJ. Comics and Medicine: Peering Into the Process of Professional Identity Formation. *Academic Medicine*. 2015;**90**:774-779.

8. Lemay M, Encandela J, Sanders L, Reisman A. Writing Well: The Long-Term Effect on Empathy, Observation, and Physician Writing Through a Residency Writers' Workshop. *Journal of graduate medical education*. 2017;**9**:357-360.

9. Levine RB, Kern DE, Wright SM. The impact of prompted narrative writing during internship on reflective practice: A qualitative study. *Advances in Health Sciences Education*. 2008;**13**:723-733.

10. Miller E, Balmer D, Hermann N, Graham G, Charon R. Sounding narrative medicine: studying students' professional identity development at Columbia University College of Physicians and Surgeons. *Acad Med*. 2014;**89**:335-342.

11. Wesley T, Hamer D, Karam G. Implementing a Narrative Medicine Curriculum During the Internship Year: An Internal Medicine Residency Program Experience. *The Permanente journal*. 2018;**22**.

12. Morgan S, Young M, Demers L, Pasco JC, Jindal S. Combating ageism in medical education with narrative medicine. *Gerontology & Geriatrics Education*. 2025;**46**:5-16.

13. Leijenaar E, Eijkelboom C, Milota M. “An invitation to think differently”: a narrative medicine intervention using books and films to stimulate medical students’ reflection and patient-centeredness. *BMC Medical Education*. 2023;**23**:568.

14. Stumbar SE, Phan M, Samuels M. An Exploratory Study of a Fourth-Year Narrative Medicine Elective: Promoting Strategies for Personal Well-Being and Improved Patient Care. *Southern Medical Journal*. 2023;**116**:42-45.
